# Muc2 Protects against Lethal Infectious Colitis by Disassociating Pathogenic and Commensal Bacteria from the Colonic Mucosa

**DOI:** 10.1371/journal.ppat.1000902

**Published:** 2010-05-13

**Authors:** Kirk S. B. Bergstrom, Vanessa Kissoon-Singh, Deanna L. Gibson, Caixia Ma, Marinieve Montero, Ho Pan Sham, Natasha Ryz, Tina Huang, Anna Velcich, B. Brett Finlay, Kris Chadee, Bruce A. Vallance

**Affiliations:** 1 Department of Pediatrics, Division of Gastroenterology, Child and Family Research Institute, Vancouver, British Columbia, Canada; 2 Department of Microbiology and Infectious Diseases, University of Calgary, Calgary, Alberta, Canada; 3 Department of Biology and Physical Geography, Irving K. Barber School of Arts and Sciences, University of British Columbia-Okanagan, Kelowna, British Columbia, Canada; 4 Department of Oncology, Albert Einstein Cancer Center/Montefiore Medical Center, Bronx, New York, United States of America; 5 Michael Smith Laboratories and Department of Microbiology and Immunology, University of British Columbia, Vancouver, British Columbia, Canada; Yale University School of Medicine, United States of America

## Abstract

Despite recent advances in our understanding of the pathogenesis of attaching and effacing (A/E) *Escherichia coli* infections, the mechanisms by which the host defends against these microbes are unclear. The goal of this study was to determine the role of goblet cell-derived Muc2, the major intestinal secretory mucin and primary component of the mucus layer, in host protection against A/E pathogens. To assess the role of Muc2 during A/E bacterial infections, we inoculated Muc2 deficient (*Muc2^−/−^*) mice with *Citrobacter rodentium*, a murine A/E pathogen related to diarrheagenic A/E *E. coli*. Unlike wildtype (WT) mice, infected *Muc2^−/−^* mice exhibited rapid weight loss and suffered up to 90% mortality. Stool plating demonstrated 10–100 fold greater *C*. *rodentium* burdens in *Muc2^−/−^* vs. WT mice, most of which were found to be loosely adherent to the colonic mucosa. Histology of *Muc2^−/−^* mice revealed ulceration in the colon amid focal bacterial microcolonies. Metabolic labeling of secreted mucins in the large intestine demonstrated that mucin secretion was markedly increased in WT mice during infection compared to uninfected controls, suggesting that the host uses increased mucin release to flush pathogens from the mucosal surface. Muc2 also impacted host-commensal interactions during infection, as FISH analysis revealed *C. rodentium* microcolonies contained numerous commensal microbes, which was not observed in WT mice. Orally administered FITC-Dextran and FISH staining showed significantly worsened intestinal barrier disruption in *Muc2^−/−^* vs. WT mice, with overt pathogen and commensal translocation into the *Muc2^−/−^* colonic mucosa. Interestingly, commensal depletion enhanced *C. rodentium* colonization of *Muc2^−/−^* mice, although colonic pathology was not significantly altered. In conclusion, Muc2 production is critical for host protection during A/E bacterial infections, by limiting overall pathogen and commensal numbers associated with the colonic mucosal surface. Such actions limit tissue damage and translocation of pathogenic and commensal bacteria across the epithelium.

## Author Summary

Enteropathogenic *E. coli* (EPEC) and Enterohemorrhagic *E. coli* (EHEC) are important causes of diarrheal disease and other serious complications worldwide. Despite many studies addressing the pathogenic strategies used by these microbes, how the host protects itself from these pathogens is poorly understood. A critical question we address here is whether the thick mucus layer that overlies the intestinal surface plays a role in host protection. Since EPEC and EHEC do not infect mice efficiently, we used a related mouse pathogen called *Citrobacter rodentium* to infect and compare responses between wildtype mice and Muc2-deficient mice, which are defective in mucus production. We show that Muc2-deficient mice are extremely susceptible to *C. rodentium* infection-induced mortality and disease. Muc2-deficient mice were also colonized faster and had higher pathogen burdens throughout the experiment. Resident (non-pathogenic) bacteria were found to interact with *C. rodentium* and host tissues in Muc2-deficient mice, indicating Muc2 regulates all forms of intestinal microbiota at the gut surface. Deficiency in mucus production also contributed to increased leakiness of the gut, which allowed microbes to enter mucosal tissues. Our study shows that Muc2-dependent mucus production is critical for effective management of both pathogenic and non-pathogenic bacteria during infection by an EPEC/EHEC-like pathogen.

## Introduction

The attaching and effacing (A/E) bacteria Enteropathogenic *Escherichia coli* (EPEC) and Enterohemorrhagic *E. coli* (EHEC) are major contributors to the global disease burden caused by enteric bacterial pathogens [1]. EPEC infects the small bowel causing acute watery diarrhea, fever and nausea [1], [2] and is an important cause of infant diarrheal disease in developing countries. EPEC infections lead to the deaths of hundreds of thousands of infants annually from dehydration and other complications [1], [3]. In contrast, EHEC (O157:H7) infection is associated with sporadic outbreaks across industrialized countries, due to consumption of contaminated beef or water supplies [1], [4]. EHEC colonizes the large bowel and secretes the highly cytotoxic Shiga Toxin (Stx), which can lead to severe hemorrhagic colitis and bloody diarrhea in people of all ages [5]. Children are at an additional risk of EHEC-induced Hemolytic Uremic Syndrome, a potentially fatal complication caused by Stx-mediated acute renal failure [6]. Both EPEC and EHEC are minimally invasive, as they intimately attach to the apical plasma membrane of intestinal epithelial cells via a Type 3 Secretion System (T3SS). Infection causes localized destruction (effacement) of the epithelial microvilli to form the unique A/E lesion [7]. Significant advances have been made in delineating the mechanisms of A/E lesion formation and their requirement for disease [8]; however, the factors involved in host susceptibility to and defense against A/E pathogens remain ill defined.

As EPEC and EHEC are human-specific and do not cause relevant disease in animal models [7], our understanding of innate and adaptive immunity against these pathogens has come from studying related A/E bacteria that infect other mammals. *Citrobacter rodentium* is a natural A/E pathogen of mice that infects epithelial cells lining the cecum, descending colon and rectum of the murine large bowel [7], [9]. *C. rodentium* infection leads to an acute colitis, mucosal hyperplasia, barrier disruption, and loose stools, but is resolved in 3–4 weeks in C57BL/6 mice [10]. Since *C. rodentium* uses similar virulence strategies to those employed by EPEC and EHEC to infect cells, including T3SS-mediated intimate attachment and A/E lesion formation, it is widely used as an *in vivo* model of A/E bacterial infection [10]. The *C. rodentium* model also allows for identification of the cells and mediators utilized by the host to control infections by A/E pathogens. While a robust adaptive immune response involving CD4+ T cells and B cells (via immunoglobulin G (IgG) secretion) is required for pathogen clearance [11], [12], studies have shown epithelial cells to be important in limiting *C. rodentium* colonization [13], [14]. In this regard, mounting evidence suggests epithelial-derived mucin production is an additional defense mechanism to manage enteric bacterial infections [15], [16]. Mucins are high molecular weight glycoproteins characterized by extended serine, threonine, and proline-rich domains in the protein core, which are sites of extensive *O*-linked glycosylation with oligosaccharides [17]. The mucin gene family contains 16 known members in humans that can be broadly divided into membrane bound or secretory forms [15]. The membrane-bound Muc1, which is produced by all intestinal epithelial cells, has been shown to play a role in host defense against *Campylobacter jejuni in vivo*, limiting disease and systemic spread [18]. Muc1 is also upregulated in *C. rodentium* infection [19], although its role in this infection is not known. However, membrane-bound MUC3 has been associated with decreased colonization of EPEC *in vitro* [20]. Collectively, these studies suggest that mucins may play a role in limiting the pathogenesis of A/E infections.

MUC2 (mouse, Muc2) is the major colonic secretory mucin in humans and mice [21], [22]. In contrast to other epithelial mucins in the gut, MUC2 is synthesized specifically by goblet cells of the small and large intestine [22]. These cells constitutively produce MUC2 polymers, which are densely packaged into numerous apically-stored granules, and released into the intestinal lumen to form the structural basis of the mucus–gel layer [21], [23]. This layer is a biochemically complex medium, rich in carbohydrates, antimicrobial peptides and other proteins, as well as lipids and electrolytes [23], [24]. The depth of the mucus layer varies with the region of the intestinal tract, but is thickest in the colon and rectum, reaching over 800 µm in rodents [25]. Studies have revealed that Muc2-mediated mucus formation in the mammalian colon leads to 2 distinct sublayers; an inner layer that is firmly adherent to the intestinal mucosa, and an outer layer that can be washed off with minimal rinsing [26], [27]. Interestingly, commensal bacteria heavily colonize the outer of these two layers, whereas the inner layer is virtually sterile [27]. The mechanisms underlying the formation and function of these sublayers is still under investigation; however, studies in animal models have indicated that Muc2-dependent mucus production profoundly impacts intestinal physiology, as demonstrated *in vivo* with the generation of Muc2 deficient (*Muc2^−/−^*) mice [28], which lack a mucus layer [27]. Depending on their genetic background, aged *Muc2^−/−^* mice may develop colorectal cancer [28] and/or spontaneous colitis [29]. Although the exact mechanisms that lead to these intestinal disorders are still elusive, deficiency in mucus production appears to alter the normal localization of commensal microbiota within the colon [27] as well as disrupt the mechanisms that govern epithelial [28], [30], [31] and immune homeostasis [29], [32].

Despite the role of Muc2 in regulating commensal and gut homeostasis, its role in host defense against epithelial-adherent pathogens such as A/E bacteria is not clear. *In vitro* studies have implicated MUC2 in limiting colonization of epithelial cells by EPEC [20], however the biological significance of this *in vivo* is undetermined. Indeed, considering that A/E pathogens colonize the mucosal surface and should therefore be constantly in contact with secreted Muc2, there is surprisingly little known about how these pathogens interact with Muc2 and the mucus layer *in vivo*. This is a critical question since the Muc2-dependent mucus layer is one of the first anatomical features bacteria such as A/E pathogens must encounter before reaching the intestinal epithelium [33]. Such early interactions could therefore profoundly influence the course of infection. The aim of our study was to use the *C. rodentium* model of A/E bacterial infection in Muc2-sufficient (wildtype) mice and Muc2-deficient (*Muc2^−/−^*) mice to understand how A/E bacteria interact with Muc2 and the mucus layer *in vivo*, and for the first time to assess the role of these interactions in host defense against this important class of bacterial pathogens. Our studies reveal novel yet fundamental insights into how Muc2 is used by the host to control infection by an A/E bacterial pathogen.

## Results

### *C. rodentium* penetrates the mucus layer during infection

**Figure 1:**
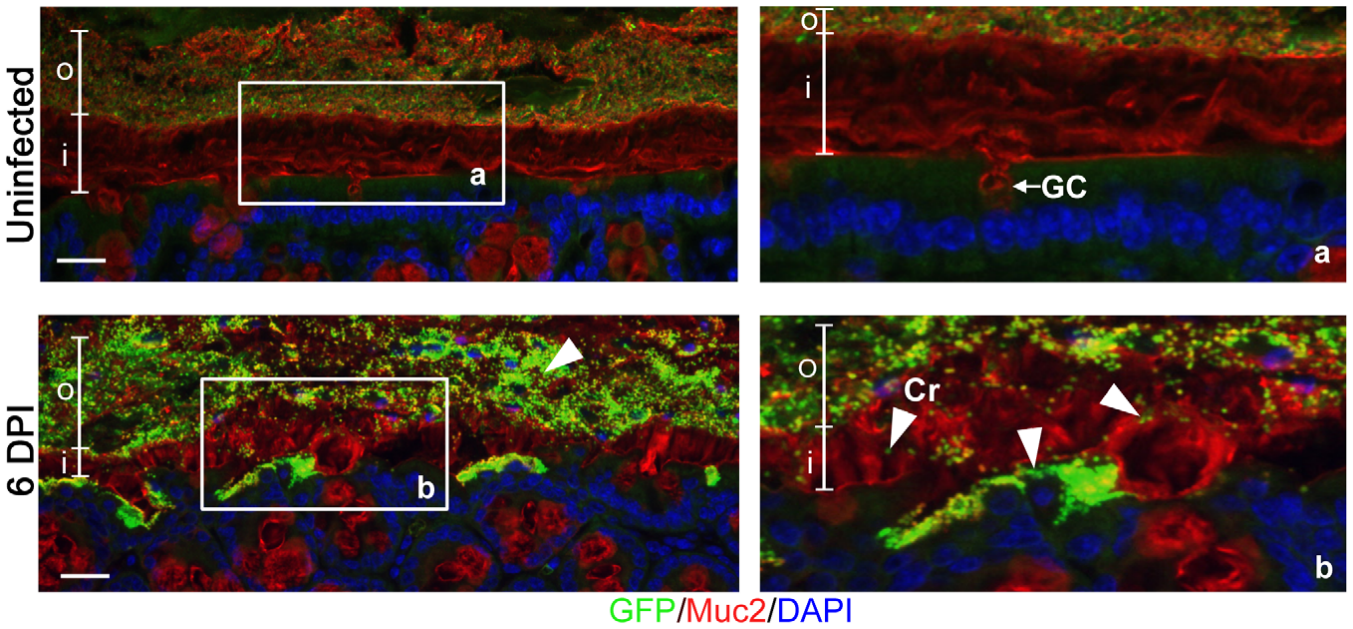
*Citrobacter rodentium* penetrates the colonic mucus layer *in vivo*. Staining for GFP-expressing *C. rodentium* using an antibody that recognizes GFP (green), and murine Muc2 (red), with DAPI (blue) as a counterstain. No GFP-labeled *C. rodentium* can be seen in the mucus layers of uninfected mice (upper panels), but in infected mice, *C rodentium* is observed within the outer and inner mucus layer in regions where the underlying epithelium is infected (bottom panels). Right panels “a” and “b” are expanded images of corresponding boxed regions in left panels. o =  outer mucus layer; i  =  inner mucus layer; Cr  =  *C. rodentium*; GC  =  goblet cell. Original magnification  = 200×. Scale bar  = 50 µm.

While *C. rodentium* is known to infect the colonic mucosal surface by directly attaching to epithelial cells, its location with respect to the colonic mucus layer has not been previously assessed *in situ*. To study this, we infected C57BL/6 mice with a green-fluorescent protein (GFP)-expressing *C. rodentium*, and at 6 days post-infection (DPI) we euthanized mice and fixed large intestinal tissues in Carnoy's fixative, which preserves the mucus layer [34]. To maximize our ability to visualize the bacteria, we conducted dual immunostaining for GFP to label *C. rodentium*, and murine Muc2 to label the inner and outer mucus layer. In uninfected tissues, no GFP-staining was observed, confirming the specificity of the GFP antibody (Figure 1, top panels). However, during infection, we found GFP-*C. rodentium* widely spread in the outer mucus layer, as well as interspersed throughout the normally sterile inner mucus layer often in proximity to infected epithelial cells (Figure 1, bottom panels). These are the first studies to definitively show *C. rodentium* within and ultimately crossing both colonic mucus layers *in situ*. Since *C. rodentium* is able to circumvent the mucus barrier, we sought to more clearly define whether this Muc2-rich layer actually protects the host, by infecting mice genetically deficient in Muc2.

### Muc2-deficient mice exhibit heightened susceptibility to *C. rodentium* infection

**Figure 2:**
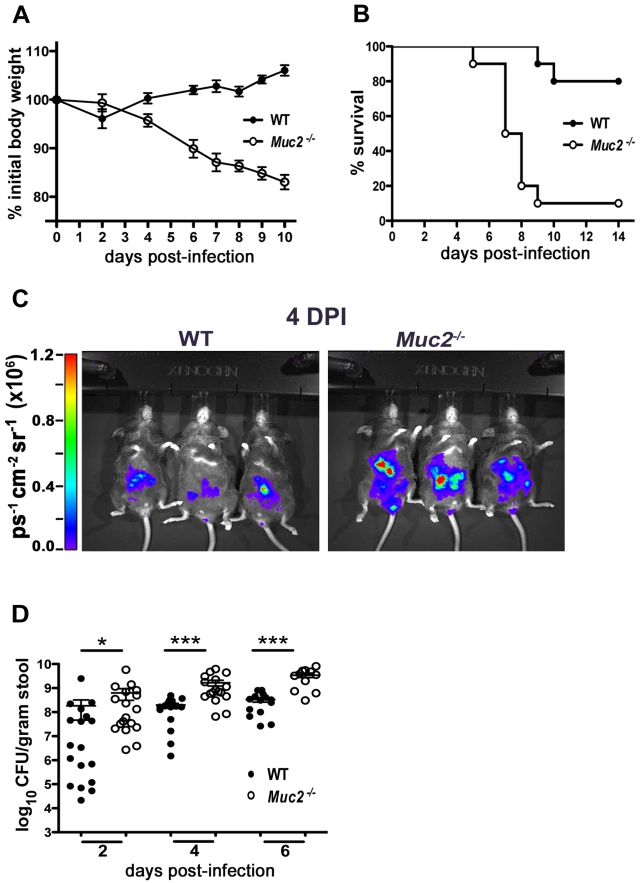
*Muc2^−/−^* mice exhibit dramatic susceptibility to *C. rodentium*-induced morbidity and mortality. **A.** Body weights following *C. rodentium* infection of WT (n = 10) and *Muc2^−/−^* (n = 10) mice. *Muc2^−/−^* mice rapidly lose weight following *C. rodentium* infection. Results are representative of 2 independent experiments. **B.** Survival curve of WT mice (n = 10) and *Muc2^−/−^* mice (n = 10) following *C. rodentium* infection. Results are representative of 3 independent infections, each with 5–10 mice per group. **C.** Bioluminescent imaging showing WT and *Muc2^−/−^* mice at 4 DPI with a luciferase-expressing *C. rodentium*. The color bar is displayed on the left where red corresponds to the highest signal intensity and blue corresponds to the lowest signal intensity, with corresponding logarithmic units of light measurement (photons s^−1^ cm^−2^ seradian^−1^). Overall signal was significantly greater by 3–10 fold in the *Muc2^−/−^* mice vs. WT mice (*P = 0.039, students *t*-test, 3 mice per group). **D.** Enumeration of *C. rodentium* in stool at various times post-infection. Each data point represents one animal. Results are pooled from two separate infections. (2 DPI, *P = 0.013; 4 DPI, ***P<0.0001; 6 DPI, ***P = 0.0004, Mann-Whitney test).

We first infected C57BL/6, *Muc2^+/+^* mice and *Muc2^−/−^* mice with *C. rodentium* and monitored body weights and survival over the first 2 weeks of infection. Since we did not detect any significant phenotypic differences between C57BL/6 and *Muc2^+/+^* mice following infection, we will subsequently refer to these mice as wildtype (WT) mice. As shown in Figure 2A, infected WT mice displayed a slight drop in weight at 2 DPI, followed by recovery and a progressive weight gain over the following week. In contrast, *Muc2^−/−^* mice steadily lost weight as their infection progressed. By 6 to 10 DPI *Muc2^−/−^* mice had lost on average over 15% of their initial body mass (Figure 2A). This was associated with several clinical signs of morbidity, including hunched posture, bloody diarrhea, and inactivity, to the point where they became moribund and had to be euthanized. Ultimately, depending on the infection, 80–100% of *Muc2^−/−^* mice required euthanization, compared to only 0–20% of WT mice (Figure 2B).

We hypothesized that Muc2 secretion and mucus layer formation would limit *C. rodentium* colonization. Therefore, to address whether the mortality suffered by *Muc2^−/−^* mice was associated with increased *C. rodentium* burdens, we monitored bacterial levels first via bioluminescent imaging of live mice using a luciferase-expressing *C. rodentium* [35]. Significantly stronger overall signals (3 to 11 fold) were observed emanating from the abdomens of *Muc2^−/−^* mice at 4 DPI. (Figure 2C). To verify this by another method, we conducted colony counts on stool samples from mice following oral infection with a streptomycin-resistant strain of *C. rodentium*. Our results showed significantly increased levels of *C. rodentium* in the stools of infected *Muc2^−/−^* mice, at levels 10 to 100 fold those found in WT mice starting at 2 DPI, and this significance was maintained at 4 and 6 DPI (Figure 2D). Thus, *Muc2^−/−^* mice were colonized at a faster rate and to a greater extent than WT mice.

### *Muc2* ^−/−^ mice exhibit worsened mucosal damage and microcolony formation on their mucosal surface

**Figure 3:**
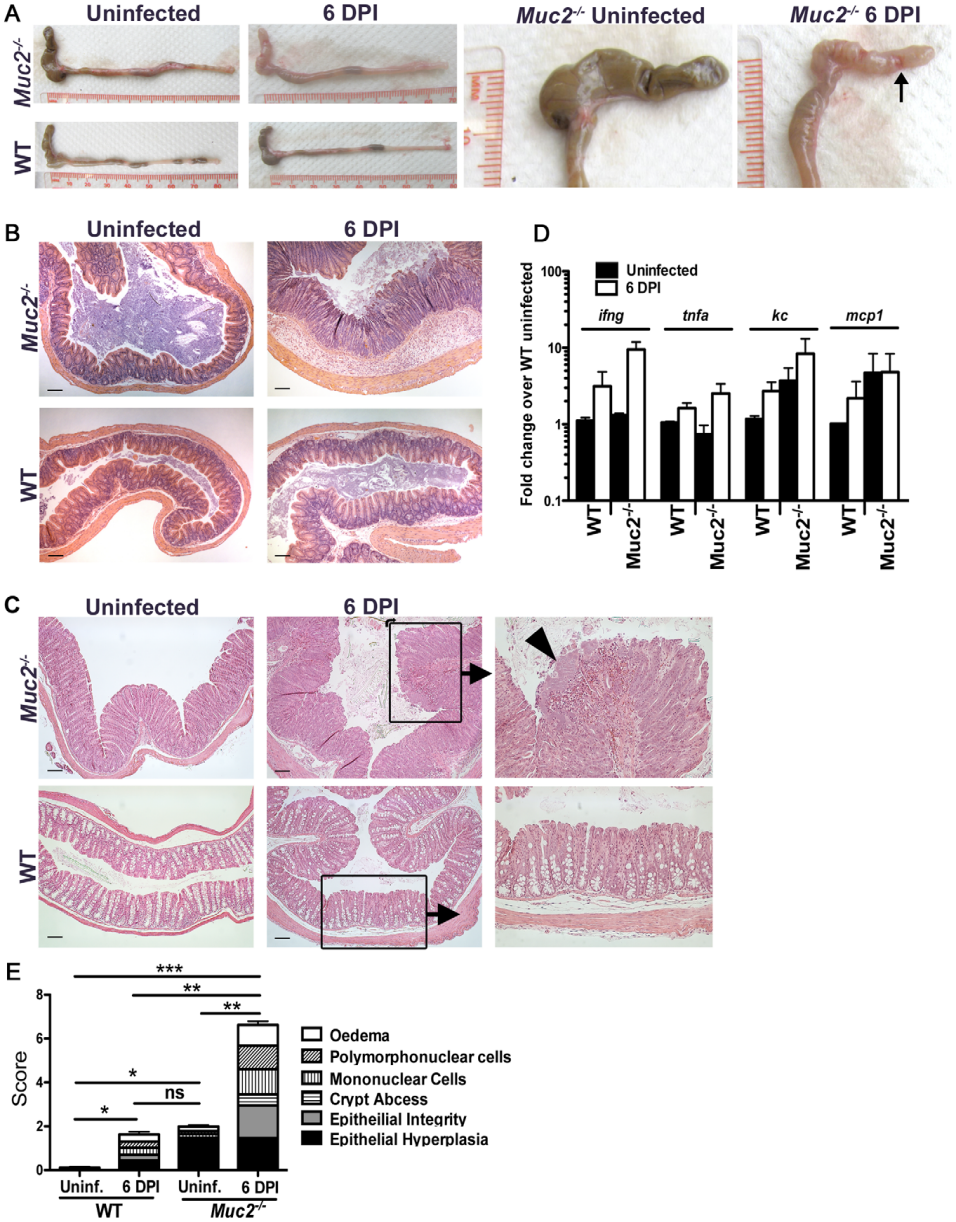
Heightened mucosal damage in *Muc2^−/−^* mice is associated with increased pathogen burdens and mucosa-associated bacterial overgrowths. **A.** Resected large intestines of uninfected and infected WT and *Muc2^−/−^* mice at 6 DPI. Note the shrunken, inflamed cecum of *Muc2^−/−^* mice compared to uninfected *Muc2^−/−^* mice, as well as the focal ulcers (arrow, right panel). **B.** H&E stained cecal sections from uninfected and infected WT and *Muc2^−/−^* mice at 6 DPI. Inflammation is found throughout the mucosa and submucosa of *Muc2^−/−^* mice (top right panel). Original magnification  = 100×. Scale bar  = 100 µm. **C.** H&E stained sections of descending colons from uninfected and infected WT and *Muc2^−/−^* mice at 6 DPI. Diffuse damage is associated with the mucosa of infected *Muc2^−/−^* mice. *C. rodentium* microcolonies can be seen associated with the mucosa in regions of ulceration (arrowhead, top right panel). Original magnification  = 100×. Scale bar  = 100 µm. **D.** Quantitative PCR results of pro-inflammatory chemokine and cytokine gene expression analysis in the ceca of uninfected or infected mice. Results represent the mean of the averages from 3 independent infections, each with 2–4 mice per group. Error bars  =  SEM. **E.** Cumulative histologic damage scores from colorectal tissues of WT vs *Muc2^−/−^* mice under uninfected and infected conditions. Scores were determined by two independent observers under blinded conditions. Results represent the means of 3–9 experiments with 2–4 mice per group. Error bars  =  SEM (*P<0.05, **P<0.005, *** *P<*0.0001, Students t test).

Concomitant with the increased bacterial burdens were overt signs of worsened macroscopic damage to the large intestines of infected *Muc2^−/−^* mice. This was characterized macroscopically by a shrunken cecum, which in approximately (≈) 60% of mice exhibited focal ulcerations (Figure 3A, arrow, right panel). There was thickening of the descending colon and rectum (colorectal tissue) of infected *Muc2^−/−^* mice (Figure 3A left panels), and in ≈40% of mice ulcers were also observed in these regions. Histological analysis of H&E stained sections confirmed the exaggerated damage in the infected *Muc2^−/−^* mice: In the cecum there was marked submucosal edema, extensive regions of mucosal hyperplasia, and increased cellular infiltrate throughout the cecal wall (Figure 3B, upper right panel). Similar features were seen in the descending colon and rectum; however, although edema was less overt, there was diffuse damage to the surface mucosa, including ulceration in this region (Figure 3C). The inflammatory cell infiltrate consisted primarily of neutrophils and macrophages as assessed by myeloperoxidase (MPO) and F4/80 staining, respectively (Figure S1A). In contrast, only minimal pathology and reduced inflammatory cell recruitment was observed in infected WT mice (Figure 3A–C; Figure S1A).

The increased damage in infected *Muc2^−/−^* mice correlated with enhanced expression of genes encoding inflammatory markers including keratinocyte-derived cytokine (KC), monocyte chemoattractant protein-1 (MCP-1), interferon-gamma (IFN-γ) and tumor necrosis factor-alpha (TNF-α) primarily in the cecum (Figure 3D), and in the colon (Figure S1B). We also assessed the expression of genes encoding colitis-associated cytokines that influence susceptibility to *C. rodentium* infection, including IL-17A and IL17F [36], IL-22 [37], and IL-23 [38]. The levels of these cytokines were upregulated to a similar degree in infected WT and *Muc2^−/−^* compared to uninfected WT mice (Figure S1C). Additionally, the IL-22-regulated lectin regenerating islet-derived III-gamma (RegIII-γ) which can prevent *C. rodentium*-induced mortality in susceptible mice [37], was also highly upregulated in both strains during infection, and elevated at baseline in uninfected *Muc2^−/−^* mice (Figure S1C). Although the large intestinal inflammatory tone (i.e. inflammatory gene expression) of *Muc2^−/−^* mice was elevated at baseline relative to uninfected WT mice (Figure 3D, Figure S1B and C), this did not translate to any overt inflammatory cell infiltrate or mucosal damage as determined by histopathological scoring (Figure 3E); however it was accompanied by increased colonic crypt lengths compared to WT mice, as was previously reported [28] (Figure 3C upper left vs. lower left panel), giving rise to the higher score in uninfected *Muc2^−/−^* vs. WT mice (Figure 3E). Overall, following infection, histological damage scores were significantly higher in *Muc2^−/−^* mice compared to all other groups (Figure 3E).

During our histological examinations, we also noticed focal aggregation of *C. rodentium* on the mucosal surface of colorectal tissues in *Muc2^−/−^* mice, giving rise to bacterial microcolonies, similar to those described by Bry and Brenner [39]. These *C. rodentium* microcolonies were frequently seen overlying ulcerated mucosal regions (Figure 3C, upper right panel), which were highly populated with neutrophils in direct contact with the microcolonies (Figure S1D). The ulcers also contained macrophages and necrotic epithelial cells (not shown). These microcolonies and ulcers were not observed in infected WT mice (Figure 3C, bottom right panel).

### Muc2 deficiency renders mice more susceptible to attenuated *C. rodentium* strains, although susceptibility is T3SS dependent

**Figure 4:**
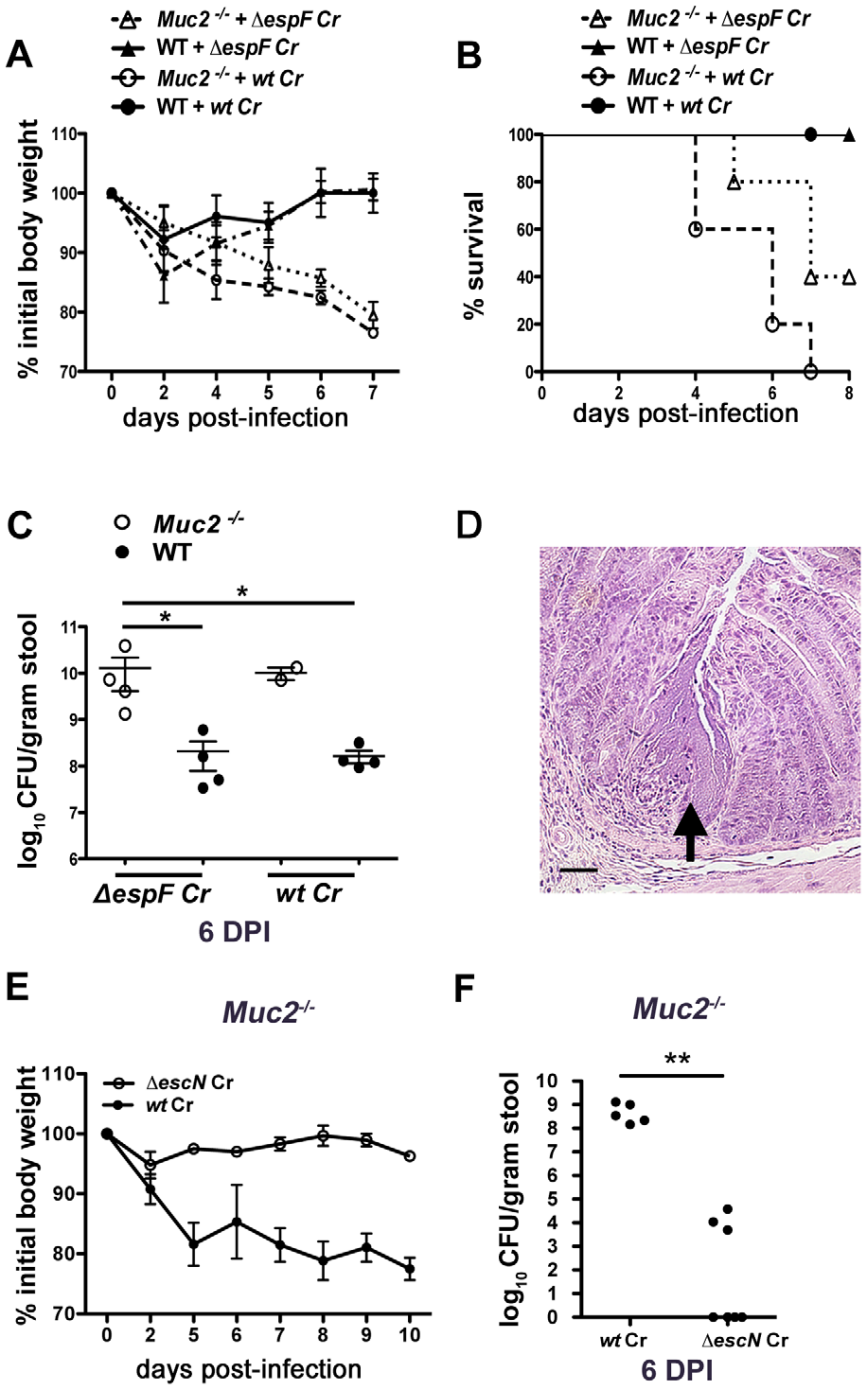
Muc2 deficiency renders mice more susceptible to attenuated strains, but susceptibility is T3SS dependent. **A.** Body weights following infection of WT and *Muc2^−/−^* mice with *wt* or *ΔespF C. rodentium*. n = 5 mice per group. Error bars  =  SEM. **B.** Survival curve of *wt* or *ΔespF C. rodentium* infected WT (n = 5) and *Muc2^−/−^* mice (n = 5). *ΔespF C. rodentium* infection results in comparable mortality to that of *wt C. rodentium* in *Muc2^−/−^* mice. **C.** Assessment of fecal burden of *wt* or *ΔespF C. rodentium*. Each data point represents the value from one individual. Error bars  =  SEM (*Muc2^−/−^* + *ΔespF Cr* vs. WT + ΔespF Cr, ^*^P = 0.0286; *Muc2^−/−^* + *ΔespF Cr* vs. WT + wt Cr, ^*^P = 0.0286, Mann-Whitney test). **D.** Representative H&E staining of colorectal section from *ΔespF C. rodentium*-infected *Muc2^−/−^* mice. Arrow points to *ΔespF C. rodentium* microcolony on an ulcerated mucosal surface. Original magnification  = 200×. Scale bar  = 50 µm. **E.** Analysis of body weights of *wt* or *ΔescN C. rodentium* infected *Muc2^−/−^* mice. Results are representative of 2 independent infections, with 2–3 mice per group. **F.** Assessment of fecal burdens of mice in E. Results are pooled from 2 individual experiments with 2–3 mice per group (**P = 0.005, Mann-Whitney test).

We next asked whether the mucosal injury occurred through previously described virulence mechanisms. *C. rodentium*, as well as other A/E pathogens, is known to cause epithelial injury and apoptosis primarily through the actions of the translocated effector EspF [40], [41]. This effector plays a critical role in causing ulcerations in other susceptible mouse strains [42], so we infected both WT and *Muc2^−/−^* mice with wildtype (*wt*) or *ΔespF C. rodentium*. As expected, the *wt* and *ΔespF* mutant caused minimal morbidity to WT mice as assessed by measurement of weight loss (Figure 4A). In contrast, there was significant weight loss in the *Muc2^−/−^* mice infected with *ΔespF C. rodentium* that was associated with 60% mortality rate, although there was a delay in the onset of these phenotypes compared to *wt C. rodentium* infection (Figure 4B). Moreover, consistent with these results, there were higher fecal *ΔespF C. rodentium* burdens in *Muc2^−/−^* mice compared to WT mice (Figure 4C). Interestingly, histology revealed that the *ΔespF C. rodentium* strain also formed the same microcolonies as *wt C. rodentium*, in concert with focal mucosal ulcerations underlying these overgrowths (Figure 4D). These data indicate that these microcolony-associated ulcerations develop independently of the translocated effector EspF.

To further test the degree of susceptibility of these mice, we infected them with a *C. rodentium* strain, *ΔescN*, which is unable to form a functional T3SS and is therefore severely impaired in virulence [43], [44]. In contrast to the *ΔespF* mutant, *ΔescN C. rodentium* failed to induce weight loss in *Muc2^−/−^* mice, or colonize it to any significant degree (Figure 4E&F). Collectively these results show that Muc2-deficiency renders mice more susceptible to even attenuated A/E bacterial pathogens; however the susceptibility does not extend to strains lacking a functional T3SS.

### Muc2 limits initial colonization of the mucosal epithelia, but ultimately controls the levels of luminal bacteria loosely associated with the mucosal tissue

**Figure 5:**
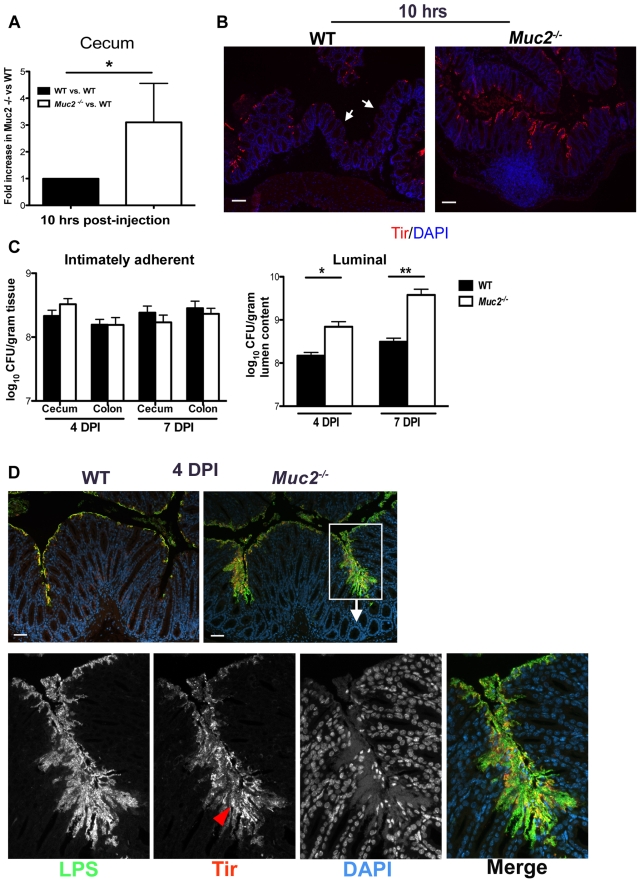
Muc2 limits initial colonization of the mucosal epithelia, but ultimately controls levels of luminal pathogen burdens. **A.** Fold differences of intimately adherent *C. rodentium* numbers present in the ceca of WT vs *Muc2^−/−^* mice 10 hours post-injection of 1.5×10^8^ CFU into cecal lumen in a cecal loop surgery experiment (see Material & Methods). Results are of data from a total of 5 mice per group pooled from 2 individual experiments. Error bars  =  SEM (*P = 0.0109, Mann-Whitney test). **B.** Representative immunostaining for the *C. rodentium-*specific effector Tir in ceca acquired from cecal loop surgery, 10 hrs post-injection. *C. rodentium* is found on the surface of *Muc2^−/−^* cecal mucosa in a continuous fashion compared to WT mice, where Tir staining is patchy amid long stretches of uncolonized surface epithelium (white arrows). Original magnification, 100×. Scale bar  = 100 µm. **C.** Quantification of luminal *C. rodentium* vs. intimately adherent *C. rodentium* attached to the cecal and colonic mucosa in WT vs. *Muc2^−/−^* mice at 4 and 7 DPI. Results represent the mean value pooled from 2 independent infections containing 3–4 mice per group. Error bars  =  SEM (*P = 0.0140; **P = 0.005, Mann-Whitney test). **D.** Visualization of *C. rodentium* infection by staining for LPS (green) and Tir (red; red arrowhead), with nuclei specific DAPI (blue).as a counterstain. Tir staining is localized to the surface epithelium in both WT and *Muc2^−/−^* mice indicating direct infection, but the majority of LPS-positive cells in *Muc2^−/−^* mice are not infecting (Tir-negative), yet are accumulating on the surface of the mucosa. Original magnification, 200×. Scale bar  = 50 µm.

While our histological stains confirmed that *C. rodentium* crosses the mucus layer to infect the underlying epithelium, the analysis of fecal burdens suggested that Muc2 limits *C. rodentium* colonization of large bowel epithelium. Consistent with this idea, *in vitro* studies have shown that rabbit mucins can inhibit EPEC attachment to epithelial cells in culture [45]. These data collectively suggest mucus may play a role in innate host defense by acting as a physical barrier to limit pathogen access to the epithelium. We tested this using an *in vivo* colonization assay. This was performed through cecal loop surgery in WT and *Muc2^−/−^* mice, where the ascending colon was tied off with sutures and 1×10^8^ *C. rodentium* were injected into the cecum (see also Materials and Methods). Ten hrs later, when the mice were euthanized and the ceca were removed, thoroughly washed of their contents, homogenized and plated, we found significantly greater numbers of adherent bacteria attached to the ceca of *Muc2^−/−^* mice compared to WT mice (Figure 5A). These counts were supported by immunostaining for the *C. rodentium*-derived infection marker translocated intimin receptor (Tir) [46], where a greater mucosal surface area was positive for Tir in the *Muc2^−/−^* ceca, compared to WT ceca that exhibited only patchy Tir staining (Figure 5B). These results demonstrate that Muc2 production limits the rate of intestinal epithelial colonization by this A/E pathogen *in vivo*.

Despite these findings, it was unclear if a doubling in the colonization rate, as seen in the cecal loop model could explain the 10–100 fold increase in total pathogen burdens found in the orally infected *Muc2^−/−^* mice. We therefore quantified intimately adherent (i.e. directly infecting epithelial cells) versus luminal (non-infecting) *C. rodentium* in the cecal and colorectal tissues of orally infected WT and *Muc2^−/−^* mice, focusing on 4 and 7 DPI, prior to when *Muc2^−/−^* mice become moribund. Unexpectedly, we found no significant difference at either time point in the number of intimately adherent *C. rodentium* in the large bowel of *Muc2^−/−^* mice compared to WT mice (Figure 5C). However there was a significant and dramatic 10-fold increase in the numbers of luminal *C. rodentium* recovered from *Muc2^−/−^* mice compared to WT mice (Figure 5C).

To clarify what these burdens meant with respect to how *C. rodentium* interacted with the mucosa *in situ*, we stained for *C. rodentium* lipopolysaccharide (LPS) as well as the infection marker Tir. Immunostaining at 4 DPI showed that in both strains, *C. rodentium* primarily infected the mucosal surface (Tir-positive), but did not invade the crypts (Figure 5D). Interestingly, while there was significantly more LPS staining in *Muc2^−/−^* tissues, most of the staining was focused in patches where large numbers of *C. rodentium* accumulated on the mucosal surface, although only a small fraction of these bacteria expressed Tir and were thus directly attached to and infecting the epithelium (Figure 5D, bottom panels). These results indicate that Muc2 deficiency does not significantly impact the total number of bacteria that ultimately infect the tissue, but predisposes the large bowel to greater numbers of loosely (i.e. non-epithelial) adherent bacteria on the mucosal surface, giving rise to the increased overall luminal burdens. As the infection progressed to 6 DPI, when mice started to become moribund, it appeared that the microcolonies were more invasive, as they penetrated deeper into the crypts and were more frequently associated with ulcerated regions (not shown, and Figure 3). Thus the propensity to accumulate bacteria on the surface of a Muc2-deficient mucosa is likely a key contributory factor to the ulcer development that occurs in these mice during infection.

### The increased luminal *C. rodentium* burdens in *Muc2* ^−/−^ mice are not due to intrinsic defects in antimicrobial activity at their mucosal surface

**Figure 6:**
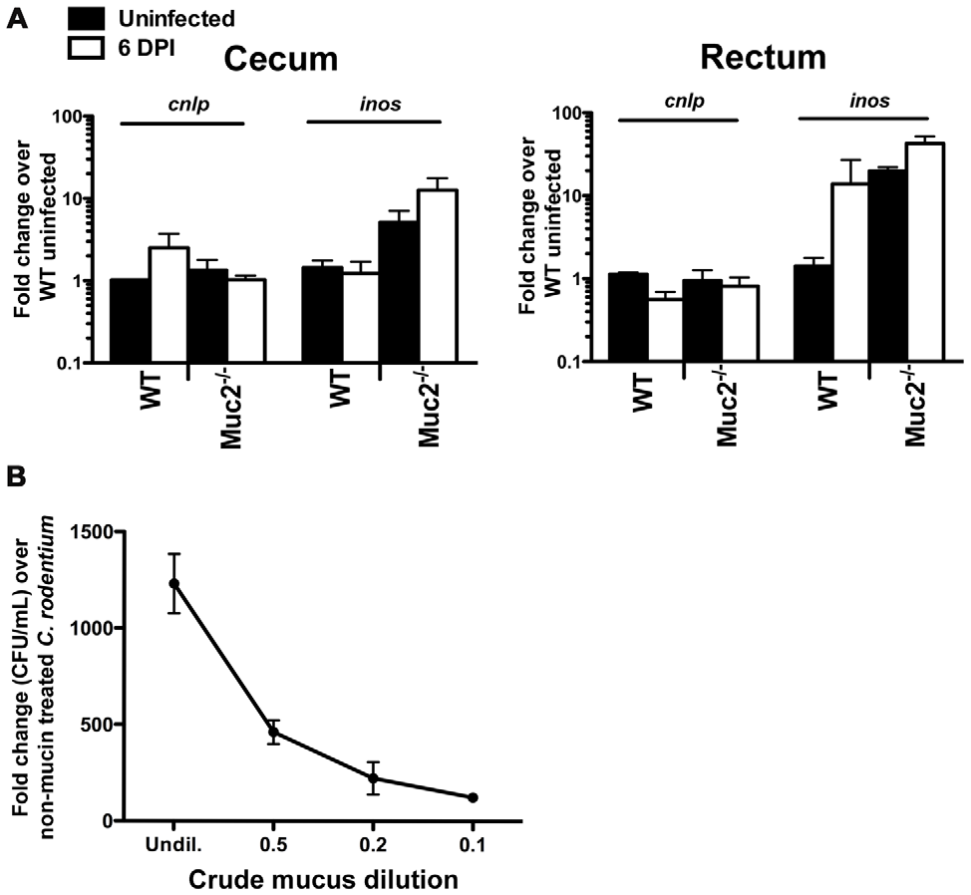
Evidence that *Muc2^−/−^* mice do not have intrinsic defects in anti-microbial activity at their mucosal surface. **A.** Quantitative PCR analysis of *cnlp* (encodes mCRAMP) and *inos* expression in the cecum and rectal tissues of WT and *Muc2^−/−^* mice. Results represent the means from 3 independent infections, each with 2–3 animals per group. Error bars  =  SEM. **B.** Titration curve from a microtitre assay showing crude mucin isolated from colorectal tissues of WT mice contains dose-dependent growth activity on *C. rodentium*. Assay was performed in duplicate for each dilution. Error bars  =  SEM. Results are representative of 2 independent experiments.

We have shown that the mucus layer provides a structural barrier that limits initial *C. rodentium* attachment *in vivo*; however, this barrier effect does not readily explain the accumulation of loosely adherent bacteria and microcolony formation at the mucosal surface of *Muc2^−/−^* mice. One plausible explanation for these overgrowths is an overall reduction in antimicrobial activity at the epithelial surface. To assay antimicrobial production in *Muc2^−/−^* mice, we first looked at the gene expression levels for epithelial-derived murine cathelicidin-related antimicrobial peptide (mCRAMP) and inducible nitric oxide synthase (iNOS), which have been shown to play a role in controlling *C. rodentium* levels *in vivo* [13], [47]. We did not see any significant differences in the expression of *cnlp* (mCRAMP), between mouse strains however, and the expression of *inos* was higher in *Muc2^−/−^* mice (Figure 6A). These data were supported at the protein level by immunostaining (not shown), indicating that the loss of Muc2 does not result in overt defects in the expression or production of innate factors known to control this pathogen.

An alternative explanation could be that Muc2 is essential for controlling pathogen numbers on the colonic surface by mediating direct antibacterial activity as shown for gastric mucus against *Helicobacter pylori* [44], and/or indirect activity by acting as a matrix to strategically position host defense peptides, as recently shown for small bowel mucus [43]. To address this in the large bowel, we tested the antimicrobial activity of crude mucus isolated from the colorectal tissues of WT uninfected mice, in a manner similar to that described by Meyer-Hoffart *et al.* [48]. Interestingly, we found no evidence that the crude colonic mucus had any antimicrobial activity against *C. rodentium*; instead, the addition of the mucus actually led to increased *C. rodentium* growth, likely by acting as a nutrient source (Figure 6B).

### Mucus secretion is increased in response to *C. rodentium* infection

**Figure 7:**
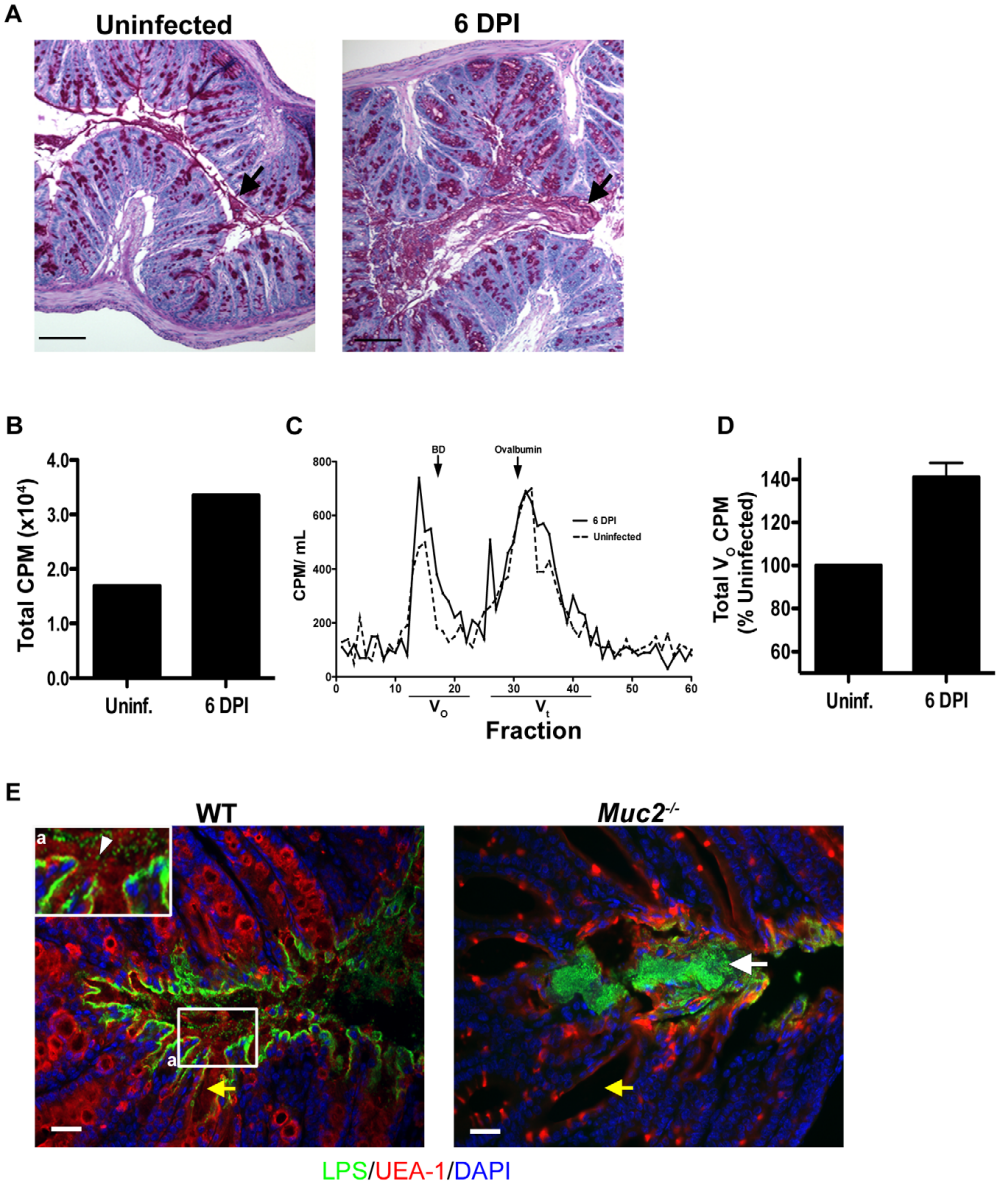
*C. rodentium* infection results in increased mucin secretion during infection. **A.** Representative PAS/Haematoxylin staining of Carnoy's fixed rectal sections from uninfected (left panel) and *C. rodentium*-infected mice (right panel). Arrow points to luminal mucus. Original magnification  = 100×. Scale bar  = 100 µm. **B.** Total counts per minute (CPM) of [^3^H]-glucosamine labeled glycoproteins found in colorectal secretions 3.5 hrs post-injection from uninfected and infected (6 DPI) WT mice. Results are representative of 2 independent infections containing 5 mice per group. **C.** Plot of liquid scintillation counts of fractions containing [^3^H] activity after total secretions were subjected to gel filtration on a Sepharose 4B chromatography column. This graph is representative of 2 independent infections with 5 mice per group. **D.** Graph of total CPMs of void volumes of S4B-fractionated mucins as described in D. Data represents the mean of the average of 2 independent experiments, each with 5 mice per group. Error bars  =  SEM. **E.** Combined epifluorescent staining for mucus using the lectin UEA-1 (red), and *C. rodentium* LPS (green), and cellular DNA (blue) using DAPI as a counterstain in heavily infected (6 DPI) regions of the colorectal mucosa from WT and *Muc2^−/−^* mice, as indicated. Individual *C. rodentium* (arrowhead, inset “a”) can be seen in mucus overlying a single layer of *C. rodentium* on the mucosal surface of a WT mouse. A *C. rodentium* microcolony (white arrow) can be seen in vicinity of a Muc2/mucus-deficient environment as indicated by the absence of mucus in the crypt lumens in *Muc2^−/−^* mice compared to WT mice (yellow arrow). Original magnification  = 200×. Scale bar  = 50 µm.

In the absence of antimicrobial activity by the mucus layer, another mechanism by which Muc2 could limit luminal numbers of *C. rodentium* is by binding to and mechanically flushing *C. rodentium* out of the colon. It has already been shown that intestinal mucus binds with high affinity to pathogens [49] including *C. rodentium* [19], and that bacterial products [50] as well as host factors stimulate mucin release both *in vitro* and *in vivo* [51]. Therefore, we hypothesized that enhanced mucus secretion could be key to the rapid removal of loosely adherent *C. rodentium* from the mucosal surface. To determine if we could see evidence of this histologically, we first conducted periodic acid-Schiff (PAS) staining on Carnoy's-fixed colorectal sections from uninfected and *C. rodentium*-infected mice at 6 DPI. As shown in Figure 7A, infected WT mice showed evidence of increased luminal mucus staining compared to uninfected mice.

To quantify this increased mucus production, we conducted pulse-chase experiments using [^3^H]-glucosamine injections in mice to metabolically label glycoproteins such as mucins in uninfected and infected mice. Mucin secretion was analyzed at 6 DPI when bacteria exhibit uniform colonization of the distal colorectal mucosa. At 3.5 hrs post-injection of [^3^H]-glucosamine, we extracted total secretions from the entire colon of control and infected mice, and quantified the secretions via scintillation counting. We observed ≈30% higher total counts per minute (CPM) in secretions from infected vs. uninfected mice (Figure 7B). To determine how this related to mucin vs. non-mucin production, we subjected the [^3^H]-labeled secretions to fractionation on a Sepharose 4B column calibrated with blue dextran (fractions 17–22), and ovalbumin (fractions 30–35) where mucins are eluted in the void volumes (V_o_) and non-mucin glycoproteins are eluted in later fractions (V_t_) [52]. Graphical analysis of the fractions (Fraction # vs. CPM), revealed a higher amplitude and larger breadth of the peak of the V_o_ fractions (#13–21) of D6-infected mice compared to uninfected controls (Figure 7C). This translated to an average 40±10% increase in [^3^H]-labeled mucin in the pooled high molecular weight V_o_ fractions in infected mice (Figure 7D).

To visualize how mucus secretion specifically impacts host-pathogen interactions, we conducted dual epifluorescent staining for *C. rodentium* LPS and *Ulex europaeus* agglutinin UEA-1, which binds to fucosylated residues abundant in mucus. Staining was performed on colorectal tissues at 6 DPI in WT mice in heavily infected regions where Muc2/mucus responses were underway. Supporting and extending the findings of previous reports [19], [44] we identified a single layer of *C. rodentium* infecting the epithelium, with no signs of microcolony formation. Instead numerous individual *C. rodentium* were seen intermixed within the luminal mucus directly above but not in contact with intimately adherent bacteria (Figure 7E, left panel and inset). In stark contrast, when we conducted UEA-1/LPS staining in *Muc2^−/−^* mice (6 DPI) we found that, although there were UEA-1 positive hypotrophic goblet cells, the crypt lumens were devoid of mucus as expected, and the absent mucus was replaced by a *C. rodentium* microcolony on the surface epithelium (Figure 7E, right panel). These results strongly suggest that secretion of mucus is important for removing loosely associated bacteria from the mucosal surface.

Although Muc2 is the major secreted mucin in human and mouse colon under baseline and inflammatory conditions [27], [53], [54], other intestinally expressed mucins may also contribute to the secreted mucin pool. We assessed the gene expression of several mucins that have been implicated in *C. rodentium* infection, and/or that are up-regulated in colitis, including the cell-surface mucins Muc1 and Muc3/17, and Muc13 [19], and the secreted non-gel forming mucin Muc4 that can be expressed by goblet cells [19], [55]; we also looked mucins that have gel-forming capacity, including the secreted gel-forming salivary and gastric mucins Muc19 [56] and Muc6 [57] respectively. There were no major changes in any of these mucins except for Muc6, which was elevated in *Muc2^−/−^* mice at baseline and also increased in WT mice during infection relative to uninfected WT controls (Figure S2A). However, because PAS staining revealed a virtual absence of mucin-filled phenotypically distinct goblet cells, and luminal mucus, under uninfected and infected conditions in *Muc2^−/−^* mice compared to WT mice, Figure S2B), this suggests that the expression of other mucins, particularly secreted gel forming mucins, do not compensate for the loss of Muc2 during *C. rodentium* infection.

### Muc2 secretion regulates commensal and pathogen numbers in the large bowel lumen

**Figure 8:**
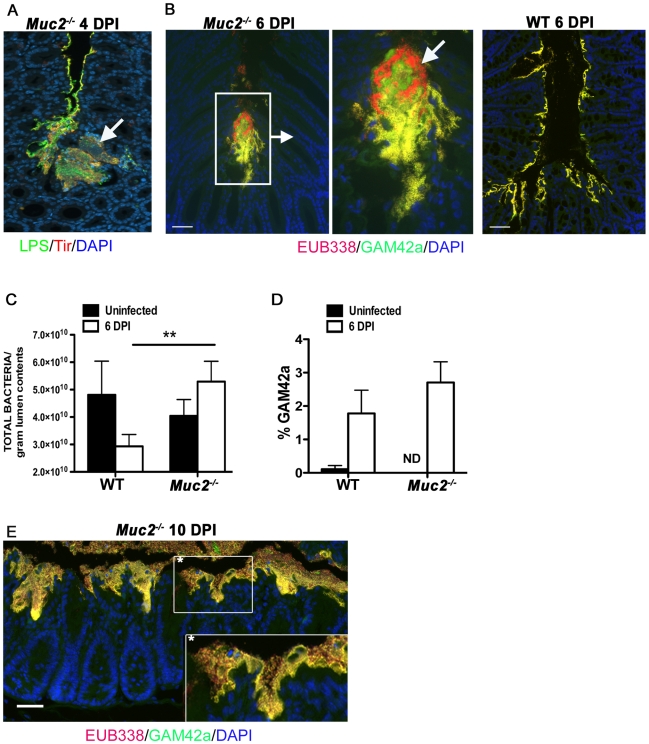
Increased luminal load of both pathogenic and non-pathogenic bacteria in *Muc2^−/−^* mice during infection. **A.** Immunofluorescence staining for *C. rodentium* LPS and DAPI in *Muc2^−/−^* at 4 DPI Notice DAPI-stained bacteria that are negative for LPS in the *C. rodentium* microcolonies (arrow). Original magnification  = 200×. **B.** Dual FISH staining using DNA probes that label virtually all true bacteria (EUB338, red) and the γ-Proteobacter class to which *C. rodentium* belongs (GAM42a, green). Pathogenic bacteria (i.e. EUB338^+^/GAM42a^+^ cells) are yellow, and commensal bacteria (EUB338^+^/GAM42^−^) cells are red. Note the non-ulcer associated bacterial microcolony containing commensal bacteria (red) mixed in with pathogenic bacteria (yellow) in *Muc2^−/−^* mice (left panels). Such mixed microcolonies were not seen in WT mice, which show predominantly pathogenic bacteria intimately adherent to the mucosa (right panel). Tissues were fixed in Carnoy's fixative prior to processing. Original magnification  = 200×. Scale bar  = 100 µm. **C.** SYBR green quantification of total bacterial burden per gram of colorectal lumen contents of WT vs. *Muc2^−/−^* mice before infection and at 6 DPI. Results are presented as the means of a total of 5–7 mice per group pooled from 2 independent experiments. Error bars  =  SEM (**P = 0.0082, Mann-Whitney test). **D.** Graph illustrating the percent composition of γ-Proteobacter (EUB338^+^/GAM42a^+^ cells), which is primarily represented by *C. rodentium*, in colorectal luminal content from uninfected or infected WT vs. *Muc2^−/−^* mice. Results are the mean percentages from a total of 5–7 mice per group pooled from 2 independent experiments. ND, none detected. Error bars  =  SEM. **E.** FISH staining as described above, showing a thick biofilm of mostly pathogenic but also commensal bacteria on the mucosal surface in a colonic section from a moribund *Muc2^−/−^* mouse at 10 DPI (inset). Such phenotypes were not observed in WT mice. Original magnification  = 200×. Scale bar  = 50 µm.

Uninfected *Muc2^−/−^* mice have been shown to exhibit commensal bacteria interacting with their mucosal surfaces more frequently than WT mice [27]. Interestingly, following staining for *C. rodentium* LPS within the microcolonies, we noted numerous LPS-negative bacteria intermixed with the positively staining bacteria (Figure 8A), suggesting these microcolonies contained other bacterial species in addition to *C. rodentium*. To test this we conducted dual fluorescence in situ hybridization (FISH) staining on colorectal sections of infected *Muc2^−/−^* mice as well as WT mice after infection using a Texas-Red conjugated EUB338 probe that recognizes 99% of all bacteria, as well as an AlexaFluor 488-conjugated GAM42a probe that detects γ-Proteobacter, the class to which *C. rodentium* belongs [58]. Our results show that in regions of microcolony formation in infected *Muc2^−/−^* mice, the majority of bacteria were EUB338**^+^**GAM42a**^+^** (*C. rodentium*, yellow), but there were distinct clusters of EUB338**^+^**GAM42a^–^ (commensal, red) bacteria mixed in with the EUB338**^+^**GAM42a**^+^** cells, confirming that these microcolonies contain non-*C. rodentium* bacterial species (Figure 8B, left panels). Moreover, numerous commensal species could be seen interacting with the epithelium in other regions (not shown). In contrast, in WT mice (6 DPI) the epithelial surface was primarily colonized with EUB338**^+^**GAM42a**^+^** cells as expected (Figure 8B, right panel); and while scattered EUB338**^+^**GAM42a^–^ bacteria were occasionally seen in the luminal mucus or near the surface, we did not observe them forming microcolonies with *C. rodentium* or interacting with the mucosal surface as we observed in *Muc2^−/−^* mice.

The above results suggest that if Muc2 promotes host defense by flushing *C. rodentium* away from the mucosal surface and out of the colon, then most enteric microbes, including commensals, would be affected by such a response. Recent studies have shown that *C. rodentium* induced colitis causes dramatic, host-mediated changes in the commensal bacterial communities in the murine colon, including a significant reduction in total commensal numbers [58]. To test whether Muc2 plays a role in this response we measured bacterial numbers within the colorectal lumen via SYBR green staining in uninfected and infected WT and *Muc2^−/−^* mice. Our results show comparable bacterial densities in the colons of uninfected WT and *Muc2^−/−^* mice (Figure 8C). During infection of WT mice, the density of total luminal bacterial numbers began decreasing over the course of infection, with a ≈40% reduction evident by 6 DPI, consistent with the findings of Lupp *et al.* [58]. In contrast, there was a ≈30% increase in the total luminal bacteria recovered from *Muc2^−/−^* mice, a density significantly greater than that recovered from WT mice (Figure 8C). Analysis of the colorectal luminal contents revealed that although the percent composition of γ-Proteobacter, most of which are *C. rodentium* [58], [59], in the *Muc2^−/−^* mice was slightly greater compared to WT mice (Figure 8D) the vast majority (97%) of the bacteria in both mouse strains were commensals. Thus, *Muc2^−/−^* mice do not undergo the commensal loss seen in the WT mice, and in fact, exhibit a trend toward increased numbers compared to uninfected controls, although this was not significant. As the infection progressed up to 10 DPI in the *Muc2^−/−^* mice, FISH staining revealed that the mucosa became covered with a thick biofilm of pathogenic microbes mixed in with commensal bacteria (Figure 8E), which was never observed in WT mice. These results collectively suggest that during infection, Muc2 plays a critical role in regulating both pathogen and commensal interactions at the mucosal surface.

### Exaggerated barrier disruption and translocation of pathogenic and commensal bacteria in infected *Muc2^−/−^* mice

**Figure 9:**
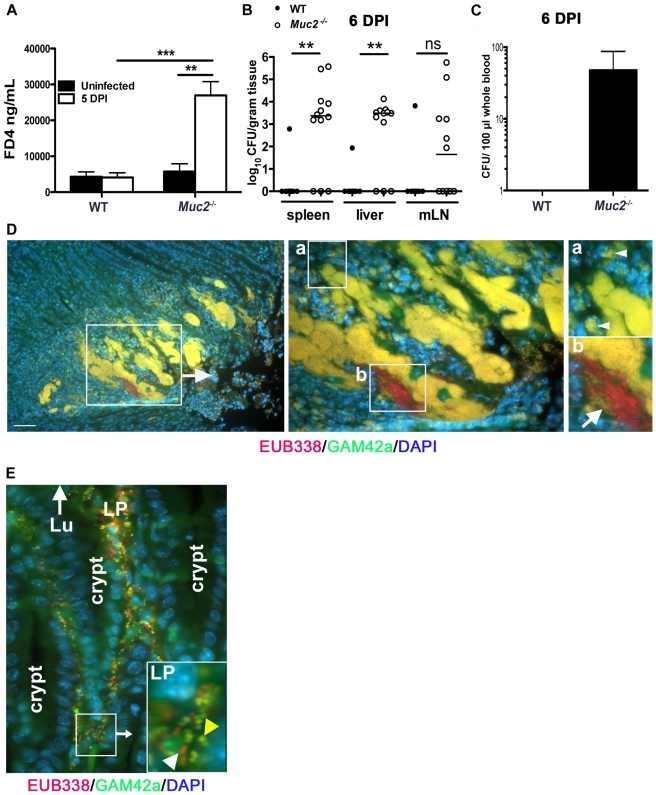
Susceptibility of *Muc2^−/−^* mice to *C. rodentium* is associated with severe defects in intestinal barrier function and increased translocation of commensal and pathogenic bacteria. *Muc2^−/−^* mice display increased FITC-dextran flux across the intestinal mucosa during *C. rodentium* infection. Uninfected or *C. rodentium* infected (5 DPI) WT and *Muc2^−/−^* mice were gavaged with FITC-dextran (4 kDa) and serum was taken by cardiac puncture 4 hrs later, as described in Materials and Methods. **A.** Quantity of FD4 in serum from WT and *Muc2^−/−^* mice. Bars represent the average value of a total of 5–7 mice per group pooled from 2 individual experiments. Error bars  =  SEM (**P = 0.0051; ***P = 0.0006, Mann-Whitney test). **B.** Quantification of viable *C. rodentium* found in the spleens, liver, and MLNs of WT and *Muc2^−/−^* mice at 7 DPI. Each data point represents one animal. Bars represent the means of 9 WT and 12 *Muc2^−/−^* mice pooled from 3 independent experiments. Error bars  =  SEM (**P = 0.0031, Mann-Whitney test). **C.** Enumeration of live bacterial burdens cultured from the serum of *Muc2^−/−^* and WT mice at 6 DPI. Results represent the average of 8 WT and 12 *Muc2^−/−^* mice pooled from 3 independent experiments. Error bars  =  SEM. **D.** FISH staining showing invasive microcolonies within an ulcerated region in the descending colon of an infected *Muc2^−/−^* mouse. Pathogenic bacteria can be seen engulfed by PMNs that are attacking the microcolony (inset “a”, arrowheads). A commensal bacterial microcolony (red) can also be seen amongst the *C. rodentium* microcolonies and in contact with PMNs (inset “b”, arrow). Original magnification  = 200×. Scale bar  = 100 µm. **E.** Numerous γ-Proteobacter (*C. rodentium*, yellow; yellow arrowhead in inset) and non-γ-Proteobacter (red; white arrowhead in inset) can be seen invading the lamina propria of infected *Muc2^−/−^* mice (6 DPI). Lu =  gut lumen. LP =  lamina propria; Original Magnification, 200×. Results are representative of 3 separate experiments.

Next, we examined the factors potentially responsible for the high mortality rates seen in infected *Muc2^−/−^* mice. We speculated that the increased numbers of luminal and surface-associated bacteria would not on their own cause the deaths of *Muc2^−/−^* mice, however the association of the loosely-associated overgrowths with superficial ulceration (Figure 3C) suggested that infection-induced epithelial barrier disruption and bacterial translocation might play a causal role in their mortality. To assess this potential, we infected WT and *Muc2^−/−^* mice and at 5 DPI we orally gavaged the mice with fluorescein isothiocyanate (FITC)-Dextran (4 kDa) (FD4) and assessed the translocation of FD4 from the gut lumen into the serum. Our results showed a striking and significant increase in the amount of FD4 in the serum of infected *Muc2^−/−^* mice compared to infected WT mice and uninfected *Muc2^−/−^* mice (Figure 9A). These results demonstrate that *C. rodentium* infection leads to a dramatic increase in intestinal permeability in the absence of Muc2. As expected, we saw similar results in response to *ΔespF C. rodentium* (not shown). To determine whether the exaggerated barrier disruption seen in *Muc2^−/−^* mice led to greater systemic pathogen burdens, we analyzed systemic sites, including the spleen, liver and mesenteric lymph nodes (MLNs) at 6 DPI. We found significantly higher *C. rodentium* burdens in the spleen, liver, and a trend toward higher burdens in the MLNs in infected *Muc2^−/−^* vs. WT mice (Figure 9B). We also found consistently higher colony forming units (CFUs) of *C. rodentium* isolated from whole blood of *Muc2^−/−^* mice that was plated directly after cardiac puncture (Figure 9C).

Since increased commensal numbers were observed loosely associated with the epithelial surface, we examined their interactions with the damaged tissue by FISH as above. When we stained the ulcerated regions, we observed EUB338**^+^**GAM42a^–^ (commensal) bacteria interacting with numerous invasive EUB338**^+^**GAM42a**^+^** (*C. rodentium*) microcolonies, and both were found amidst a dense population of polymorphonuclear leukocytes (PMNs) (Figure 9D). Numerous bacteria were also seen within the cell bodies of PMNs (Figure 9D, insets “a” and “b”). At times of barrier disruption, large numbers of both *C. rodentium* and non-γ-Proteobacter species could be found deep within the mucosa of infected *Muc2^−/−^* mice (Figure 9E). Rarely if ever were microbes observed in the mucosa of infected WT mice. These results strongly suggest that both pathogenic and commensal bacteria contribute to the disease and mortality suffered by *Muc2^−/−^* mice, since A/E bacterial infection-induced disruption of the epithelial barrier allows massive translocation of both pathogenic and commensal bacteria out of the intestinal lumen and into mucosal tissues, and pathogens into systemic compartments, leading to bacteremia.

### Evidence that Muc2-deficiency reduces host-mediated pathogen clearance when commensal-dependent host colonization resistance is compromised

**Figure 10:**
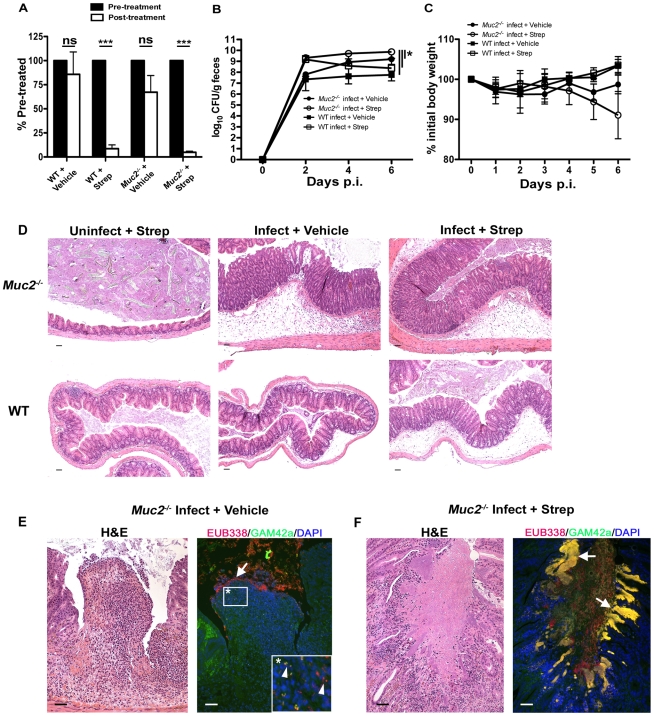
Antibiotic induced commensal depletion enhances pathogen colonization but does not alter host pathology in *Muc2^−/−^* mice. **A.** Quantification of DAPI stained bacteria from stools of WT and Muc2−/− mice 24 hours following oral treatment with Streptomycin (20 mg) or Vehicle (dH_2_0). Streptomycin (strep) led to significantly reduced numbers of total bacteria within mouse stool. Results represent the means of 3–4 mice per group. Error bars  =  SEM (***P<0.001, unpaired t test). **B.** Enumeration of *ΔespF C. rodentium^Str^* (strep-resistant) in stool of strep-or vehicle-treated mice as indicated, at various times post-infection. Results represent the means of 3–4 mice per group. Error bars  =  SEM (*P≤0.05, Mann-Whitney test, one-tailed). **C.** Body weights following infection of strep or vehicle treated WT and *Muc2^−/−^* mice with *ΔespF C. rodentium^Str^*. n = 3–4 mice per group. Error bars  =  SEM. **D.** Representative histological sections of ceca from uninfected or infected (6 DPI) strep- or vehicle-treated WT and *Muc2^−/−^* mice. Original magnification  = 100×. Scale bar  = 100 µm. **E.** H&E (Left panel) and FISH analysis (right panel) of an ulcer from *ΔespF C. rodentium^Str^* infected vehicle-treated *Muc2^−/−^* mouse cecum (6 DPI). Numerous commensals (EUB338^+^/GAM42^−^ cells, red) can be seen overlying the ulcer in direct contact with PMNs (arrow), and both pathogen (EUB338^+^/GAM42a^+^ cells, yellow) and commensal (red) can be seen within the PMNs (arrow heads, inset). Original magnification  = 200×. Scale bars  = 100 µm. **F.** H&E and FISH analysis of an ulcer in the descending colon from an *ΔespF C. rodentium^Str^* infected strep-treated *Muc2* ^−/−^ mouse (6 DPI). Large pathogenic microcolonies (yellow) are associated with the ulcer (arrows), while commensals (red) can be seen in the lumen. Original magnification  = 200×. Scale bar  = 100 µm.

The above data show commensal and pathogenic bacteria occupying intestinal niches in *Muc2^−/−^* mice that are not colonized in WT mice during infection. To attempt to elucidate the precise role of commensal bacteria during *C. rodentium* infection in *Muc2^−/−^* mice, we administered a high dose of the antibiotic streptomycin (20 mg/mouse) by oral gavage to reduce the numbers of total commensals prior to infection. Stool was collected immediately prior to treatment and again 24 hrs later, and then stool bacteria was quantified as above to confirm commensal depletion. Streptomycin (strep) treatment resulted in a significant (average 10–20 fold) reduction in commensal bacterial numbers in both WT and *Muc2^−/−^* mice, while vehicle treatment did not cause any significant changes (Figure 10A). Neither treatment led to any inflammation or pathology on its own when assessed 7 days later (not shown). 24 hrs after treatment, strep- and vehicle-treated WT and *Muc2^−/−^* mice were also gavaged with *ΔespF C. rodentium^Str^* (strep-resistant), which was chosen instead of *wt C. rodentium* because it is less virulent. Colonization was assessed by plating stool contents every second day. The results show that at 2 DPI, strep-treated WT and *Muc2^−/−^* mice carried 10–50 fold higher bacterial burdens compared to infected vehicle-treated WT and *Muc2^−/−^* mice (Figure 10B). However by 4 and 6 DPI, while *ΔespF C. rodentium^Str^* burdens began to decline in infected strep-treated WT mice ultimately to levels similar to infected vehicle-treated WT mice (6 DPI), bacterial burdens in infected strep-treated *Muc2^−/−^* mice continued to increase to levels significantly higher than all other groups (Figure 10B). Moreover, burdens in infected vehicle-treated *Muc2^−/−^* mice also increased to levels that were higher than infected strep-treated WT mice at 6 DPI. Although weight loss varied among mice both *Muc2^−/−^* groups, only WT mice tended to gain weight during infection (Figure 10C).

At 6 DPI, both cecal and colonic tissues were resected and assessed by histology. As shown by H&E (Figure 10D, bottom panels), strep-treatment led to increased edema and inflammation in WT ceca compared to vehicle-treated WT mice during infection; however in infected *Muc2^−/−^* tissues, there were no obvious differences in cecal and colonic inflammation between strep-and vehicle-treated groups (Figure 10D, top panels). Overt ulceration was seen in the ceca of vehicle-treated *Muc2^−/−^* mice (Figure 10E), while ulcers were observed in the colons of strep-treated *Muc2^−/−^* mice (Figure 10F) Interestingly, FISH staining of cecal sections from infected vehicle-treated *Muc2^−/−^* mice showed large numbers of commensals (EUB338**^+^**GAM42a^–^, red) directly interacting with PMNs in ulcerated regions (Figure 10E, left panel). These interactions were seen at the mucosal surface of ulcers where there was little evidence of *ΔespF C. rodentium*; however *ΔespF C. rodentium^Str^* could still be seen within the PMNs (Figure 10E, right panel, inset). In contrast, large invasive *ΔespF C. rodentium^Str^* microcolonies (EUB338**^+^**GAM42a**^+^**, yellow) could be seen associated with the ulcers in the colons of infected strep-treated *Muc2^−/−^* mice (Figure 10F, right panel). Such pathology was never observed in uninfected mice or in any of the infected WT groups. Collectively, these results indicate that (i) Muc2 promotes host-mediated colonization resistance when commensals are depleted; and (ii) commensal bacteria, although initially important in promoting colonization resistance in both strains, ultimately come into direct contact with large numbers of PMNs following the infection-induced ulceration that occurs in a Muc2-deficient environment. Thus Muc2 is critical for managing commensal and pathogenic bacteria within the GI tract, particularly at mucosal surfaces during an enteric infection.

## Discussion

The Muc2-rich mucus layer is the first host-defense barrier that noxious luminal agents contact in the intestine [33], and as such, it functions as the main interface between the host and its luminal microbiota. To our knowledge, this is the first study to formally demonstrate the importance of the major mucus glycoprotein Muc2 in host defense against an A/E bacterial pathogen *in vivo*. We show that the presence of Muc2 and hence the mucus layer is necessary to protect against severe mucosal damage and barrier dysfunction during infection. This was in part due to Muc2 functioning as a structural barrier to limit the rate of pathogen colonization of epithelial cells in the large bowel. However, Muc2 plays an additional role in host defense by controlling the pathogen burden that resides within the colonic lumen, primarily by removing loosely adherent bacteria and preventing bacterial accumulation and microcolony formation on the colorectal surface. The inability to effect this removal likely contributes to the severe barrier dysfunction seen in *Muc2^−/−^* mice. We provide evidence that the ability of Muc2 to control luminal bacteria is most likely attributable to increased Muc2/mucus secretion during infection, which was demonstrated through metabolic labeling of mucin glycoproteins in WT mice. Moreover, we demonstrate that the ability of Muc2 to control luminal pathogens also impacts the resident commensal microbiota, as the microcolonies seen overlying the mucosa of infected *Muc2^−/−^* mice contained both *C. rodentium* as well as commensal microbes, and both types of bacteria were seen translocating across the colonic epithelium and into the lamina propria. These results ultimately reveal Muc2 production as a critical mechanism by which the host controls exposure to both pathogenic and commensal bacteria *in vivo*.

While we assumed that A/E pathogens such as *C. rodentium* would have to interact with the mucus layer during the course of infection, we demonstrate and characterize this interaction for the first time *in situ*. We show that *C. rodentium* colonizes the outer mucus layer in high numbers, and can also be found traversing the normally bacteria-free inner mucus layer to gain access to the underlying epithelial cells. These results raise the question of how A/E pathogens manage to circumvent the mucus layer. *C. rodentium* lacks a functional flagellum and is thus non-motile [60], and therefore likely utilizes specific mucinases or glycosidases to digest mucin in order to overcome the mucus barrier, although this has yet to be formerly demonstrated. Notably, EHEC has recently been shown to secrete the metalloprotease StcE that has apparent mucinase activity [61] suggesting A/E pathogens do employ this strategy. In contrast, despite their diversity and extreme density in mammalian colon, commensal bacteria do not penetrate the inner mucus layer to any significant degree, probably because they are more adapted to the nutrient-rich luminal environment [62]. Ultimately, this suggests that colonizing the outer and inner mucus layer is a key step for the pathogenesis of A/E bacteria, therefore, the bacterial factors involved in crossing the mucus layer are likely critical for virulence.

Our studies reveal an unexpected insight into how Muc2 mediates protection. Muc2 is widely presumed to act as a physicochemical barrier to limit access to epithelial tissues by luminal pathogens [17], including pathogens such as A/E bacteria. Several lines of evidence support this, such as the demonstration of mucins inhibiting EPEC adherence in vitro [20] and our *in vivo* cecal loop colonization assay described in this report. However, since the total numbers of bacteria that ultimately infected (i.e. became intimately adherent to) the tissue was not significantly different in a Muc2 deficient environment, the role of Muc2 as a defense barrier may be of only transient importance. Rather the major function played by Muc2, at least in response to A/E bacteria, appears to be to limit luminal burdens, mainly by preventing the accumulation of pathogens that are loosely associated with the tissue. These bacteria probably arise from replication of intimately-bound pathogens, as the T3SS mutant (*ΔescN C. rodentium*) failed to efficiently colonize. This massive increase in the overall pathogen burden at the mucosal surface has important implications for downstream host responses. EPEC and EHEC both disrupt epithelial permeability *in vitro* [63], as does *C. rodentium in vivo* [64], [65]. While intimately-adherent bacteria are firmly bound to the epithelia, the non-infecting, but loosely adherent bacteria are more likely to translocate into the mucosa, particularly when faced with the mechanical pressures of dietary flow. Indeed, at times of severe barrier disruption we saw much higher systemic levels of *C. rodentium* in the *Muc2^−/−^* mice.

Although Muc2 deficiency did not ultimately impact on the numbers of intimately-adherent *C. rodentium*, there was a striking increase in intestinal permeability in *Muc2^−/−^* compared to WT mice. The susceptibility to ulcer formation in the *Muc2^−/−^* mice is probably a major contributor to the barrier dysfunction and morbidity seen in these mice since it was associated with greater systemic pathogen burdens. While the mechanisms are unclear, we suggest the accumulation of bacteria and microcolony formation on the epithelial surface in a Muc2-deficient environment is linked to either the development and/or maintenance of the ulceration, since most ulcers were associated with the microcolonies. It has been proposed that serum proteins released at ulcerated sites contribute to ulcer-associated *C. rodentium* overgrowth [66]; however the fact we saw microcolony formation also in non-ulcerated sites argues against this always being the case. Interestingly, past studies have shown that the A/E pathogen translocated effector EspF has been linked to epithelial barrier disruption [41], [67] and ulcer-associated damage [42]. However, since ulcers, microcolony formation, and barrier disruption were also seen in mice infected with the *ΔespF* strain, these data indicate that barrier disruption occurs through non-canonical pathways. We speculate that bacterial accumulation and microcolony formation at the surface adversely affects epithelial survival either directly, by producing a high local concentration of toxic metabolites; or indirectly, by causing the recruitment of large numbers of PMNs to the site of infection, where epithelial cell death is the result of collateral damage caused by neutrophils releasing cytotoxic mediators to control the infection. In fact, one can envision these microcolonies to be an overwhelming burden to recruited phagocytes, perpetuating a vicious inflammatory cycle (Figure 10). Whatever the specific role of these invasive microcolonies, they likely exacerbate the focal damage and associated barrier defects, and thus have a severe impact on morbidity in the *Muc2^−/−^* mice.

Although we attribute the majority of the pathological phenotypes in infected *Muc2^−/−^* mice to result from *C. rodentium*, one of the striking features during the course of infection was the maintenance of commensal bacteria at the mucosal surface of the *Muc2^−/−^* mice. While we also found scattered commensal bacteria overlying the epithelium before infection [27], *C. rodentium* was clearly unable to totally displace them. This led to some intriguing phenotypes, including direct intimate interactions between commensal bacteria and the pathogen, where commensals were found intermixed with *C. rodentium* clusters to create multispecies microcolonies. Critically, commensal species could also be found translocating across the mucosal surface and into the lamina propria, where they were in direct contact with PMNs at sites of microcolony-associated ulceration, even forming microcolonies of their own. We explored whether these commensal bacteria contribute to the resulting colitis by transiently depleting them using the antibiotic streptomycin. While the depletion was successful, it also led to an exaggerated pathogen burden, confirming that commensal bacteria play an important host defense role by providing colonization resistance against *C. rodentium*. Although we did not identify overt differences in the resulting pathology in *Muc2^−/−^* mice following antibiotic pretreatment, we were unable to conclude to what degree commensal translocation might play in the resulting colitis, considering the loss of commensals occurred concomitantly with increased pathogen burdens. However, the fact that infection-induced cecal ulceration in *Muc2^−/−^* mice led to large numbers of commensals that were directly interacting with PMNs points to a pathologic host-commensal interaction during infection. Therefore, while commensals are beneficial early during an infection by enhancing colonization resistance, their continued presence as the infection progresses likely plays a pathologic role. These studies are particularly interesting in light of the study by Lupp *et al*. [58] who described an overall reduction of commensal bacterial numbers after an established *C. rodentium* infection. It has been suggested this is a pathogenic strategy where pathogens exploit inflammation to suppress commensal growth and thereby reduce colonization resistance [68]. However, our findings strongly suggest that clearance of commensal microbes from the colon after an established *C. rodentium* infection may also benefit the host, by decreasing the total bacterial burden faced by the host at a time when its intestinal barriers are compromised.

**Figure 11:**
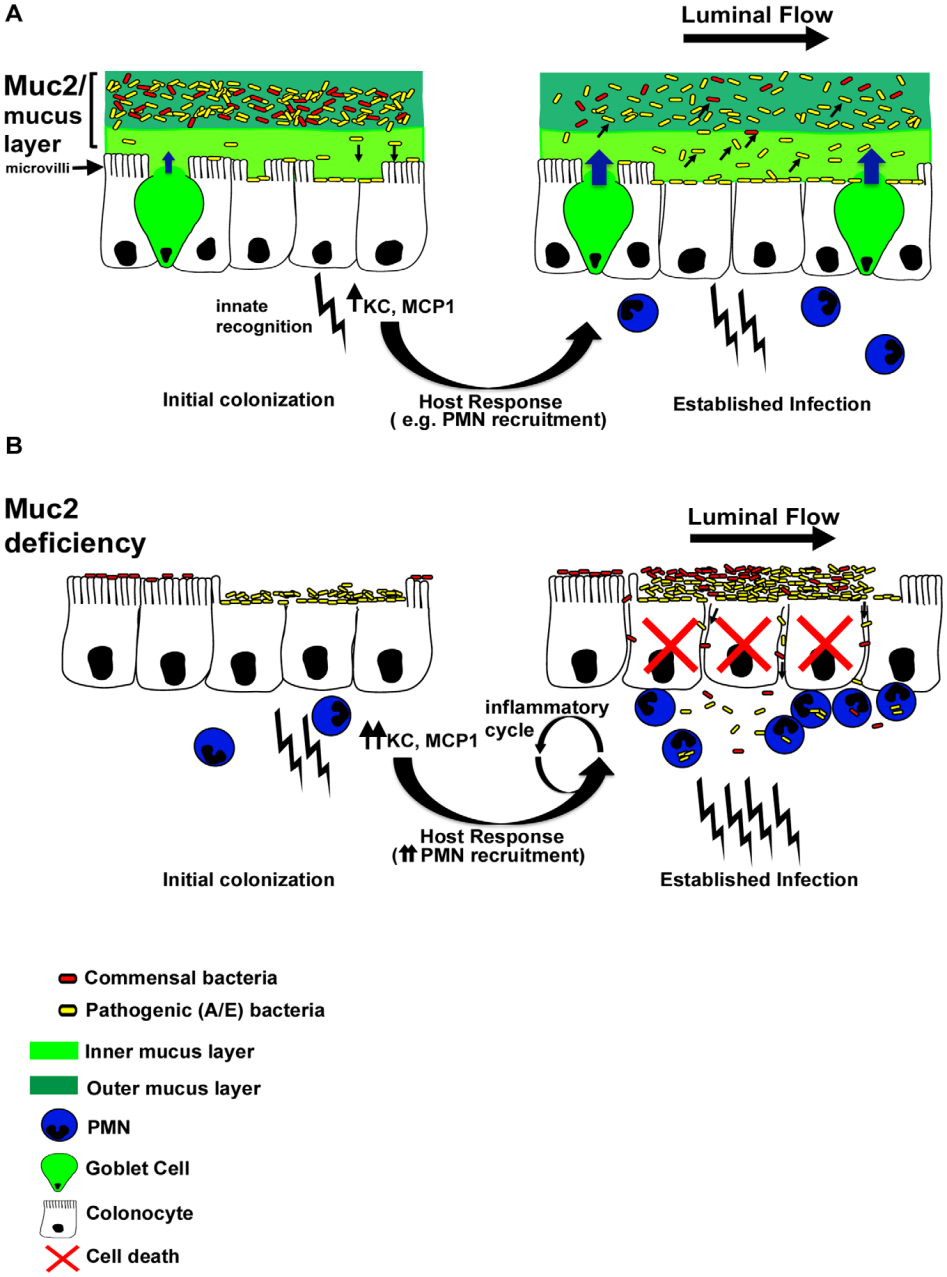
Proposed model of the role of Muc2 in the disassociation of A/E pathogen and commensal bacteria from the large intestinal mucosa. **A.** In a Muc2-sufficient intestine, A/E bacteria such as *C. rodentium* (yellow) need to first traverse the outer and inner mucus layers to access the underlying epithelium. Following infection of epithelial cells, there is an enhancement in mucin secretion probably due to synergistic actions between bacterial products and host derived cytokines after innate recognition by pattern recognition receptors, and recruitment of inflammatory cells such as PMNs. In addition, there is moderate epithelial barrier dysfunction as a result of host and pathogen induced alteration of tight junctions. As the A/E pathogen replicates following intimate attachment, the secreted Muc2 binds newly reproduced bacteria and flushes them away from the surface to prevent microcolony formation on the surface and their translocation into the mucosa. **B.** In a state of Muc2-deficiency the lack of mucus causes a more rapid infection and an accumulation of pathogens that are loosely associated with the mucosa, forming microcolonies. Commensal bacteria (red) can also be caught up in these pathogenic microcolonies, further increasing total burden at the surface and likelihood of direct and/or indirect epithelial damage. Following infection, severe barrier dysfunction occurs, mostly by altered tight junctions as well as overt epithelial cell death. As a result both the loosely-adherent pathogens and commensals leak across the epithelia and into the mucosa, overwhelming the phagocytes and perpetuating a vicious inflammatory cycle.

We hypothesize that mucin secretion is the key mechanism by which Muc2 controls the levels of A/E bacteria, and commensal bacteria at the surface. Recent studies have suggested there is enhanced mucin secretion in the colon during *C. rodentium* infection [19]. We extend these findings through metabolic labeling to show at least a 40% increase in mucus secretion in response to infection, and specifically indentified *C. rodentium* within luminal mucus. This increase in mucin secretion is likely a gross underestimate of the local increase in mucin release, since in order to have sufficient quantities for analysis, we extracted mucus from the whole colon, and the increase in secretion is expected to be focused in the descending colon and rectum where the infection occurs [69]. Due to the lack of antimicrobial activity we saw within the crude mucus, and the fact that it was recently shown by the McGuckin laboratory that *C. rodentium* directly binds to Muc2/mucus *in vitro* [19], we hypothesize that induced mucin secretion is an effective means for the host to bind and remove non-infecting, loosely adherent A/E bacteria that would otherwise accumulate on the surface and exacerbate disease (Figure 11). Although beyond the scope of the present study, an outstanding issue yet to be addressed is deciphering the precise molecules responsible for the induced Muc2 secretion *in vivo*. There are a plethora of candidates, including bacterial products, such as LPS [51], [70], or host derived cytokines such as TNFα [71], neuromodulators including vasoactive intestinal peptide [72], or neutrophils via elaboration of secretagogues such as neutrophil elastase [73], all of which have been shown to cause enhanced mucin release from goblet cells in tissue culture, and are present during *C. rodentium* infection [74], [75], [76]. Based on the data presented in our report, the elucidation of the specific host and/or microbial factors and molecular pathways that regulate mucus production during enteric bacterial infection constitutes a fertile area of research.

Importantly, while we ascribe the ability of intestinal mucus to flush away luminal bacteria from the mucosal surface to primarily reflect the actions of Muc2, there are likely other mucins, (potentially found in total secreted mucus) that may also contribute to the protective actions of the mucus. These include Muc1, a cell surface mucin that is upregulated in bacterial induced colitis [19] and potentially cleaved to release its α-subunit containing the extracellular mucin domain into the intestinal lumen, as seen during *H. pylori* infection [77]; Muc4 which can be up-regulated during DSS-induced colitis [78]and be expressed by colonic goblet cells [55]; and the secreted gel-forming mucins Muc19 and Muc6, the latter being produced in *Muc2^−/−^* mice during colitis [29]. Even so, we maintain that Muc2 is the major protective mucin. This is in part based upon the phenotype of *Muc2^−/−^* mice (confirmed by our studies), where Muc2-deficiency leads to a virtual loss of mucin-filled phenotypically mature goblet cells within the large intestine, and a corresponding loss of both the inner and outer colonic mucus layers [24] and other forms of luminal mucus. Moreover, Muc2 is by far the major secretory mucin under both baseline (in mice and humans) [24], [53] and inflammatory conditions in the colon [54]. However, we did see a modest up-regulation of Muc6 mRNA expression during infection of WT mice, and the impact of this expression is currently under investigation.

During the course of this article review, it was demonstrated by Hasnain *et al.* [79] that Muc2-deficiency renders mice more susceptible to intestinal nematode infections, suggesting Muc2 and mucus production can protect against diverse enteric pathogens. Muc2 production is clearly protective during A/E bacterial infection, but whether this is true for other enteric bacterial pathogens of the gut remains to be shown. Importantly, since bacteria and other enteric pathogens have co-evolved with their hosts, many exhibit multiple strategies to subvert and exploit host defenses including mucus to promote colonization [80]. A well known motility factor is flagella, which is commonly utilized by pathogenic bacteria such *Vibrio cholerae* to migrate through mucus (reviewed in [33]). In addition, *Salmonella* appears to anchor itself to mucus via specific adhesins [81] to promote colonization [82], and exhibits resistance to small bowel mucus antimicrobial activity [48]. *Yersinia enterocolitica* has been shown to utilize polysaccharides present in mucins like Muc2 to harvest energy and promote growth [83]. A similar observation has been shown for *Salmonella* Typhimurium, for which it has been proposed as a strategy to outcompete the commensal microbiota within an inflammatory niche [84]. Moreover, parasites such as *Entamoeba histolytica* stimulate mucin release to deplete the mucus layer [85], as well as proteolytically break down the polymeric structure of secreted Muc2 to facilitate access to the underlying epithelium [86], [87]. Thus, whether Muc2 has evolved primarily to regulate interactions with normal microbiota and other luminal contents, or to provide adequate host defense against enteric pathogens has yet to be determined. However, because the commensal microbiota is a major variable in any enteric infection, particularly in the colon, it is likely that the presence of Muc2 allows for effective immunological management of the infectious agent by limiting commensal burdens at mucosal surfaces.

In conclusion, our studies have shown that Muc2 and the mucus layer are critical for host defense against an A/E bacterial pathogen. However, it is important to note that Muc2 can potentially be modulated in several ways either during infection, such as at the level of gene expression, post-translational modification, or even at the level of secretion into the intestinal lumen. Each regulatory step may influence the biological function of Muc2, which in turn will influence how the host responds to enteric pathogens. Since Muc2 is an integral part of the colonic ecosystem, future studies are warranted to unravel precisely how intestinal mucus impacts the course of infectious disease.

## Materials and Methods

### Mice

Six to eleven-week-old C57BL/6, *Muc2^+/+^* and *Muc2^−/−^* mice (on C57BL/6 background) were purchased from the National Cancer Institute (NCI) or bred in our animal facility. Mice were kept in sterilized, filter-topped cages, handled in tissue culture hoods and fed autoclaved food and water under specific pathogen free (SPF) conditions. Sentinel animals were routinely tested for common pathogens. The protocols employed were approved by the University of British Columbia's Animal Care Committee and in direct accordance with guidelines drafted by the Canadian Council on the Use of Laboratory Animals.

### Bacterial strains and infection of mice

Mice were infected by oral gavage with 0.1 ml of an overnight culture of LB containing approximately 2.5×10^8^ cfu of *wt C. rodentium* (formerly *C. freundii* biotype 4280, strain DBS100, the EspF mutant *ΔespF C.rodentium*, or the T3SS mutant *ΔescN C. rodentium* [88]. Bioluminescent strains of *C. rodentium* were constructed by introducing plasmid pT7 (E. A. Meighen, Department of Biochemistry, McGill University) carrying the entire lux operon from *Photorhabdus luminescens*. For bacterial enumeration studies, a streptomycin-resistant derivative of *C. rodentium* DBS100 was utilized. For some studies a streptomycin-resistant *ΔespF C.rodentium* was utilized, and was constructed in our laboratory by routine procedures. GFP-*C. rodentium* was constructed within our laboratory by chromosomal insertion of *gfp* into *C. rodentium* DBS-100 via Red/ET Recombination, using a Quick & Easy *E. coli* Gene Deletion Kit (Gene Bridges) as per manufacturers instructions. The virulence of the GFP-*C. rodentium* was confirmed in preliminary studies. For commensal depletion studies, mice were pre-treated with 0.1 ml of 200 mg/ml (20 mg) streptomycin (or H_2_0) 24 hrs prior to infection.

### Tissue collection

Uninfected or infected mice were anesthetized with Halothane, killed by cervical dislocation, and their large intestines were resected and divided into cecum, ascending colon, descending colon, and rectum for further analysis. Tissues were immediately placed in 10% neutral buffered formalin (Fisher) (48 hrs, 4°C) or ice cold fresh Carnoy's Fixative (2 hrs, 4°C) or 4% paraformaldehyde (1 hr, room temp) for histological studies, or placed in RNA*later* (Qiagen) and stored at −86°C for subsequent RNA extraction.

### Bacterial counts

For enumeration of bacteria within the tissue and luminal compartments, whole mouse ceca or colons were sliced open longitudinally, and their luminal contents were collected in a preweighed 2.0 ml microtube containing 1.0 ml of phosphobuffered saline (PBS) and a 5.0 mm steel bead (Qiagen). Cecal and colonic tissues were washed vigorously in PBS (pH 7.4), cut into several pieces, and also placed in a tube as above. Tissue and lumen contents were weighed, and then homogenized in a MixerMill 301 bead miller (Retche) for a total of 6 mins at 30 Hz at room temperature. Tissue homogenates were serially diluted in PBS and plated onto luria broth (LB) agar plates containing 100 mg/ml streptomycin, incubated overnight at 37°C, and bacterial colonies were enumerated the following day, normalizing them to the tissue or stool weight (per gram). For fecal bacterial burden analysis, stool was collected from live mice at various times post-infection (described in text) and processed as described for luminal contents. For some studies with non-antibiotic resistant *C. rodentium*, plating was performed on MacConkey Agar (Difco), *C. rodentium* colonies were clearly identified by their unique characteristic of being round with red centre and a thin white rim. Colonies were confirmed to be *C. rodentium* by PCR for the *C. rodentium* T3SS translocator gene *escN*.

### Histological staining

Briefly, 5 µm paraffin sections were deparaffinized by heating at 55–65°C for 10 min, cleared with xylene, rehydrated through an ethanol gradient to water. Sections were blocked using the appropriate blocking buffer (either 2% Goat or Donkey Serum in PBS containing 1% bovine serum albumin (BSA), 0.1% Triton-X100 (Sigma), and 0.05% Tween 20, and 0.05% sodium azide. For detection of biotinylated targets, blocking of endogenous biotin was carried out prior to blocking with serum, using the Endogenous Biotin Blocking kit (Molecular Probes). Primary antibodies or lectins were diluted in PBS containing 1% BSA, 0.1% Triton-X100 (Sigma), and 0.05% Tween 20, and 0.05% sodium azide. The antibodies used were rat anti-F4/80 (1∶8000; Serotec), rabbit anti-MPO (1∶100; NeoMarkers), rat antisera generated against *C. rodentium* specific Tir (1∶5K; gift from W. Deng), rabbit anti-*E.coli* Poly 8 LPS (1∶500; Biotec Laboratories), biotinylated goat anti-GFP (1∶100: GeneTex), polyclonal antisera that recognized the murine colonic mucin Muc2 (1∶50; a gift from Jan Dekker). Staining for fucosylated mucins was carried out using biotinylated-*Ulex europaeus* agglutinin-1 (2 ug/ml; Vector Labs). Antigen retrieval was used for F4/80 and MPO staining, and was performed prior to blocking and staining by placing deparaffinized, rehydrated slides in 10 mM citric acid pH 6.0 at 90–100°C for 20 min, followed by cooling to room temperature. Preparation and staining of PFA-fixed frozen sections was performed as described previously [44]. For dual LPS/Tir staining, no detergents (TritonX-100 or Tween-20) were used in the dilution buffers, to avoid Tir staining within bacteria. Epifluorescent labeling for all stains was carried out with the appropriate secondary antibody using AlexaFluor 488-conjugated goat (or donkey) anti-rabbit IgG, AlexaFluor 568-conjugated goat anti-rabbit IgG, AlexaFluor 568-conjugated goat anti-rat IgG (all 1∶2000), or AlexaFluor 568-conjugated Streptavidin (1∶1000) (Molecular Probes/Invitrogen). Tissues were mounted using ProLong Gold Antifade reagent (Molecular Probes/Invitrogen) that contains 4′,6′-diamidino-2-phenylindole (DAPI) for DNA staining. Sections were viewed at 350, 488, and 594 nm on a Zeiss AxioImager microscope. Images were obtained using a Zeiss AxioImager microscope equipped with an AxioCam HRm camera operating through AxioVision software (Version 4.4).

### RNA extraction and quantitative RT-PCR

Colon tissues stored in RNA*later* (Qiagen) at −86°C were thawed, weighed, and total RNA extracted using the Qiagen RNeasy kit following the manufacturer's instructions. Tissues were homogenized in a 2.0 ml microtube containing 0.6 ml of Buffer RLT (supplied in Qiagen RNeasy kit) and a 5.0 mm steel bead (Qiagen), and homogenized in a MixerMill 301 bead miller (Retche) for 4 minutes at 30 Hz at room temperature. Total RNA was quantified using a NanoDrop Spectrophotometer (ND1000). 1–2 ug of RNA was reverse-transcribed using a Qiagen Omniscript RT kit (Qiagen), according to manufacturer's instructions. For quantitative PCR, cDNA was diluted 1∶5 in RNase/DNase free H_2_O and 5 µl was added to 15 µl PCR reaction mix. The final reaction volume was 20 uL, containing BioRad Supermix used at a 1∶2 dilution, and primers at a final concentration of 0.6 uM each. qPCR was carried out using a BioRad Miniopticon or Opticon2. Melting point analysis confirmed the specificity for each of the PCR reactions. Quantitation was performed using GeneEx Macro OM 3.0 software. Primer sequences and reaction conditions for or all genes analyzed are given in Table 1. All mucin primers, and Reg3g primers were designed with Primer3 (Version 0.4.0).

**Table 1:** Primer sets and PCR conditions used in this study.

Target mRNA^a^	Primer Sets	PCR cycle conditions^b^ denature/anneal/extend
IFN-γ	Fwd: 5′- TCAAGTGGCATAGATGTGGAAGAA -3′ Rev: 5′-TGGCTCTGCAGGATTTTCATG -3′	95°C, 30 s/60°C, 30 s/72°C, 30 s
TNF-α	Fwd: 5′- CATCTTCTCAAAATTCGAGTGACAA -3′ Rev: 5′- TGGGAGTAGACAAGGTACAACCC-3′	94°C, 30 s/55°C, 30 s/72°C, 45 s
KC	Fwd: 5′- TGCACCCAAACCGAAGTCAT-3′ Rev: 5′- TTGTCAGAAGCCAGCGTTCAC-3′	94°C, 30 s/57°C, 30 s/72°C, 45 s
MCP-1^c^	Fwd: 5′-TGCTACTCATTAACCAGCAAGAT -3′ Rev: 5′-TGCTTGAGGTGGTTGTGGAA -3′	94°C, 30 s/59°C, 15 s/72°C, 90 s +78°C, 5 s
iNOS	Fwd: 5′- TGGGAATGGAGACTGTCCCAG-3′ Rev: 5′- GGGATCTGAATGTGATGTTTG-3′	94°C, 30 s/60°C, 30 s/72°C, 30 s
mCRAMP	Fwd: 5′- CTTCAACCAGCAGTCCCTAGACA-3′ Rev: 5′- TCCAGGTCCAGGAGACGGTA-3′	94°C, 30 s/55°C, 30 s/72°C, 30 s
β-actin	Fwd: 5′-CAGCTTCTTTGCAGCTCCTT-3′ Rev: 5′-CTTCTCCATGTCGTCCCAGT-3′	94°C, 30 s/55–60°C, 30 s/72°C, 30 s
IL-17A	Fwd: 5′-GCTCCAGAAGGCCCTCAGA-3′ Rev: 5′-CTTTCCCTCCGCATTGACA-3′	94°C, 30 s/60°C, 30 s/72°C, 30 s
IL-17F	Fwd: 5′-TGCTACTGTTGATGTTGGGAC-3′ Rev: 5′-AATGCCCTGGTTTTGGTTGAA-3′	94°C, 30 s/55°C, 30 s/72°C, 45 s
IL-22	Fwd: 5′-ACCTTTCCTGACCAAACTCA-3′ Rev: 5′-AGCTTCTTCTCGCTCAGACG-3′	94°C, 30 s/58°C, 30 s/72°C, 30 s
IL-23p19	Fwd: 5′-TGGCTGTGCCTAGGAGTAGCA -3′ Rev: 5′-TTCATCCTCTTCTTCTCTTAGTAGATT -3′	94°C, 30 s/60°C, 30 s/72°C, 30 s
RegIII-γ	Fwd: 5′-TGCCTATGGCTCCTATTGCT-3′ Rev: 5′-CACTCCCATCCACCTCTGTT-3′	94°C, 30 s/58°C, 30 s/72°C, 30 s
Muc1	Fwd: 5′-AGGAGGTTTCGGCAGGTAAT-3′ Rev: 5′-TCCTTCTGAGAGCCACCACT-3′	94°C, 30 s/55°C, 30 s/72°C, 45 s
Muc3/17	Fwd: 5′-TGAGCAAAGGCAGTATCGTG-3′ Rev: 5′-GCCTCCTTCTTGCATGTCTC-3′	94°C, 30 s/55°C, 30 s/72°C, 45 s
Muc4	Fwd: 5′-GAAAAGCGTGTTGCCTCTTC-3′ Rev: 5′-AGAGGGAAATGCCCTGATCT-3′	94°C, 30 s/55°C, 30 s/72°C, 45 s
Muc6	Fwd: 5′-TGCATGCTCAATGGTATGGT-3′ Rev: 5′-TGTGGGCTCTGGAGAAGAGT-3′	94°C, 30 s/55°C, 30 s/72°C, 45 s
Muc13	Fwd: 5′-TCTGGACTCTGGCCACTCTT-3′ Rev: 5′-GAGGACAGAGCCAGTCCAAG-3′	94°C, 30 s/55°C, 30 s/72°C, 45 s
Muc19	Fwd: 5′-ACTGGAACCACAGCCAAATC-3′ Rev: 5′-CTACGGCCTGTTTTTCGGTA-3′	94°C, 30 s/55°C, 30 s/72°C, 45 s

^*a*^IFN-γ primers from ref. [94]; TNF-α primers, ref. [95]; MCP-1 primers, ref. [96]; mCRAMP primers, ref. [13]; iNOS primers, ref. [97]; KC primers, ref. [98]; IL-17A and IL-23p19 ref. [99]; IL-17F primers ref. [36]; and IL-22 primers ref. [100].

^*b*^All PCR experiments had an initial denaturing step of 95°C for 3–5 mins before commencement of PCR cycling conditions.

^*c*^MCP1 primers after 40 cycles had an additional 2 steps of 94°C for 30 s, and 50°C for 30 s.

### Cecal loop model

For cecal loop experiments, a 50 uL overnight inoculum of *C. rodentium* was placed in 3 mL Dulbecco's modified eagle medium and incubated without shaking at 37°C, 5% CO_2_ for 3 hrs, to induce expression of the T3SS [89]. Cecal loop experiments were modified from those previously described for ileal loop experiments [90]. In brief, mice were anaesthetized by intraperitoneal injection of ketamine and xylazine. Following a midline abdominal incision, the cecum and proximal colon were gently exteriorized, and the proximal colon at the cecal-colonic junction was ligated twice. 300 uL containing approximately 1×10^8^ cfu of pre-activated *C. rodentium* was then slowly injected into the cecal lumen. The cecum and colon were then returned to the abdominal cavity and the incision closed with discontinuous sutures. At given time points, the mice were euthanized and tissues collected for bacterial enumeration and histology as described above.

### Bioluminescent imaging

At 4 DPI with luciferase expressing *C. rodentium*, mice were anaesthetized with 2% isofluorane carried in 2% O_2_ and imaged using an IVIS (Xenogen; Almeda, CA). Greyscale reference images taken under low illumination were collected and overlaid with images capturing the emission of photons from the lux-expressing bioluminescent *C. rodentium* using LIVING IMAGE software (Xenogen) and Igor (Wavemetrics; Seattle, WA). Live mice were returned to their cages.

### Metabolic labeling

Metabolic labeling was carried out as previously described [52] with slight modifications. Uninfected (LB treated) and *C. rodentium*-infected mice were injected intraperitoneally with 20 µCi of [^3^H]glucosamine (Amersham) in 0.3 ml of Dulbeccos(D)-PBS (pH 7.2) and left for 3.5 hrs to metabolically label the large intestinal mucin pool. The animals were euthanized, and the colons were excised and flushed with PBS, and opened with fine scissors into a Petri dish and the mucosal surface was scraped with a glass slide to remove the adherent mucus. Mucosal secretions were placed in 15–20 ml of D-PBS and vortexed at high speed for 10 min, and then the supernatant was clarified by centrifugation (1,000 g for 10 min). The cell-free supernatant was reserved and glycoproteins were precipitated with equal volumes of 10% trichloroacetic acid (TCA) and 1% phosphotungstic acid (PTA) overnight at 4°C, solubilized in column buffer (8.06 mM Tris-HCL, 1.98 mM Tris- base, 0.001% sodium azide, pH 8.0) and neutralized to pH 7.0–7.4 with 0.1 mol/l NaOH. 5 ml of scintillation cocktail (UniverSol) was added, and ^3^H activity (a measure of mucus secretion) was determined in a scintillation counter. To confirm the identity of the high-molecular-weight mucin following *C. rodentium* infection, the secreted [^3^H]glucosamine-labeled glycoproteins produced in response *C. rodentium* and untreated controls were subjected to Sepharose-4B (Sigma) column chromatography. To do this, the 10% TCA-1% PTA-precipitated glycoproteins were dissolved in column buffer and applied to a S4B column previously equilibrated with 0.01 mol/l Tris HCl. Fractions (30–40 in total/0.4 ml each) were collected, and ^3^H activity of each fraction was determined by liquid scintillation counting. The results are expressed as total CPM recovered in each fraction. The column was calibrated using the following molecular weights standards: blue dextran (BD; 2,000 kDa), thyroglobulin (669 kDa) and BSA (67 kDa) (Amersham).

### FITC-dextran intestinal permeability assay

This assay was performed as previously described [75]. Uninfected or infected mice at 5 DPI were gavaged with 150 µl of 80 mg/ml 4 kDa FITC-dextran (Sigma; FD4) in PBS 4 hrs prior to sacrifice. Mice were anaesthetized and blood was collected by cardiac punctures, which was added immediately to a final concentration of 3% acid-citrate dextrose (20 mM citric acid, 100 nM sodium citrate, 5 mM dextrose) (Harald Schulze, Shivdasani Laboratory, DFCI). Plasma was collected and fluorescence was quantified using a Wallace Victor (Perkin-Elmer Life Sciences, Boston, MA) at excitation 485 nm, emission 530 nm for 0.1 s.

### Fluorescence *in-situ* hybridization

Formalin-fixed paraffin-embedded sections were deparaffinized and rehydrated as described above. Sections were incubated overnight at 37°C in the dark with Texas red-conjugated EUB338 general bacterial probe (5′-GCT GCC TCC CGT AGG AGT-3′) and an AlexaFluor 488 conjugated GAM42a probe (5′-GCC TTC CCA CAT CGT TT-3′) that recognizes bacteria that belong to the γ-Proteobacter class [58], [91] diluted to a final concentration of 2.5 ng/ul each in hybridization solution (0.9 M NaCL, 0.1 M TRIS pH 7.2, 30% Formamide, 0.1% SDS). Sections were then washed once in the dark with hybridization solution for 15 minutes with gentle shaking. This step was repeated once with wash buffer (0.9 M NaCL, 0.1 M TRIS pH 7.2), and sections were placed in dH_2_O, and then mounted using ProLong Gold Antifade reagent with DAPI (Molecular Probes) and imaged as described above. For quantification studies, the methods were carried as previously described [58].

### SYBR green DNA staining

Large intestines were collected and prepared as described above for bacterial counts, except the lumen contents from the cecum and colon were separated. After homogenization, samples were diluted 1∶10 in PBS, then 450 ul of the 1∶10 dilution was placed in 50 ul 10% Neutral Buffered Formalin, vortexed briefly, and stored at 4¡C. 2–5 ul of the 1∶10 diluted sample stored in formalin was diluted in 1 ml PBS and filtered onto Anodisc 25 filters (Whatman International Ltd) with a pore size of 0.2 µM and 2.5 cm diameter. The samples were allowed to thoroughly dry, and then were stained with 0.25 µl SYBR green (Invitrogen) in 100 µl PBS for 15 min in the dark. Alternatively, samples were filtered onto Nucleopore Track-Etch membranes (Whatman) for DAPI staining only. The filters were air dried (in the dark for SYBR staining) and mounted on glass slides with ProLong Gold Antifade reagent with DAPI (Molecular Probes) and viewed as above. The mean number of cells counted in 3 to 6 randomly chosen fields per disc was determined.

### Antimicrobial assay

Crude mucus was isolated from colorectal tissues in the same manner as described for the small intestine by by Meyer-Hoffert *et al.* [48]. Resected colons from WT mice were flushed gently with PBS using a pippette fitted to a syringe. Colons were then opened up longitudinally and placed in a Petri dish, mucosa side up. The round edge of forceps was then used to gently scrape off the inner colonic mucus layer with minimal damage to the epithelial surface. The mucus globule was placed in a tube, diluted 1∶1 with PBS, and mixed well by vortexing and pipetting up-and-down, and then immediately placed on ice. For the antimicrobial assay we conducted assays described by Turner *et al.* [92] with slight modifications. An overnight culture of streptomycin-resistant *C. rodentium* grown in LB was diluted 1∶1000 in Tryptic Soy Broth (TSB) and grown to mid log phase (OD_620_ 0.6–1.0). The bacteria was washed by centrifugation (3000 rpm, 4°C, 10 mins) and removing the supernatant, and resuspending the pellet in ice cold 10 mM sodium phosphate buffer (SPB) (pH 7.4). This step was repeated once. The washed sample was diluted to a final OD_620_ of 0.7, diluted 1000×, and 5 uL of this dilution (containing ≈1×10^4^ bacteria) were added to 25 ul 10 mM SPB with 0.03% TSB containing 50 ug/ml streptomycin +/−20 ul of various dilutions of crude mucin as described in the text. For negative controls, only SPB + streptomycin was added. The total reaction volume was 50 ul. Cultures were left for 3 hrs at room temp, then serially diluted and plated on LB plates containing 50 ug/ml streptomycin, and incubated overnight at 37°C incubator. Colonies were counted the next day.

### Histopathological scoring

To assess tissue pathology, we used a scoring system adapted from previously described scoring systems [88], [93]. In brief, paraffin-embedded colonic tissue sections (5 µm) that had been stained with haematoxylin and eosin were examined by two blinded observers. Tissue sections were assessed for submucosal edema (0 =  no change; 1 =  mild; 2 =  moderate; 3 =  profound), epithelial hyperplasia (scored based on percentage above the height of the control where 0 =  no change; 1 = 1–50%; 2 = 51–100%; 3 = >100%), epithelial integrity (0 =  no change; 1 = <10 epithelial cells shedding per lesion; 2 = 11–20 epithelial cells shedding per lesion; 3 =  epithelial ulceration; 4 =  epithelial ulceration with severe crypt destruction); neutrophil and mononuclear cell infiltration (0 =  none; 1 =  mild; 2 =  moderate; 3 =  severe). The maximum score that could result from this scoring was 15.

### Statistical analysis

Statistical significance was calculated by using either a two-tailed Student's t-test or the Mann-Whitney test unless otherwise indicated, with assistance from GraphPad Prism Software Version 4.00 (GraphPad Software, San Diego California USA, www.graphpad.com). A *P* value of ≤0.05 was considered significant. The results are expressed as the mean value with standard error of the mean (SEM).

### Gene accession numbers

The following are the GeneIDs (Database: Entrez Gene) for each gene analyzed in this manuscript, given as gene name (official symbol **GeneID #**): TNF-α (Tnf **GeneID:** 21926); IL-23p19 (Il23a **GeneID:** 83430); IFN-γ (Ifng **GeneID:** 15978); IL-17A (Il17a **GeneID:** 16171), IL-17F (Il17f **GeneID:** 257630); IL-22 (Il22 **GeneID:** 50929); MCP-1 (Ccl2 **GeneID:** 20296); KC (Cxcl1 **GeneID:** 14825); iNOS (Nos2 **GeneID:** 18126) mCRAMP (Camp **GeneID:** 12796); Muc1 (Muc1 **GeneID:** 17829), Muc2 (Muc2 **GeneID:** 17831); Muc3/17 (Muc3 **GeneID:** 666339); Muc4 (Muc4 **GeneID:** 140474); Muc6 (**GeneID:** 353328); Muc13 (Muc13 **GeneID:** 17063); and Muc19 (Muc19 **GeneID:** 239611).

## Supporting Information

Figure S1 Characterization of the inflammatory cell infiltrate within the colons of *C. rodentium*-infected WT and *Muc2^−/−^* mice. **A.** Immunostaining for infiltrating macrophages via F4/80 staining (top panels) and neutrophils via MPO staining (bottom panels) in descending colons of WT and *Muc2^−/−^* and mice. Original magnification  = 200×. Scale Bar  = 50 µm. **B.** Quantitative PCR analysis of pro-inflammatory chemokines and cytokines in the descending colons of WT and *Muc2^−/−^* mice at 6 DPI compared to their respective uninfected controls. Results averaged from 3 independent infections, with n = 2–4 mice per group. Error bars  =  SEM. **C.** Quantitative PCR analysis of genes that are associated with host-susceptibility to *C. rodentium* in the colons of WT and *Muc2^−/−^* mice at 6 DPI. Results are averaged from 4–5 mice per group, pooled from 2 independent infections. Error Bars  =  SEM. **D.** MPO staining as above in an ulcerated region of an infected *Muc2^−/−^* mouse, showing a dense population of neutrophils in direct contact with a large microcolony of *C. rodentium* (white asterisk, *C. rodentium* aggregate; arrowhead, MPO positive cell in indirect contact with the microcolony). Original magnification  = 200×. Scale Bar  = 50 µm.
(8.58 MB TIF)

Figure S2 Analysis of *Muc* family gene expression and overall mucin content in colorectal tissues of uninfected or *C. rodentium*-infected WT and *Muc2^−/−^* mice. **A.** Quantitative PCR analysis of expression of genes encoding various Muc family members in the rectal tissues of WT and *Muc2^−/−^* mice under uninfected or infected (6 DPI) conditions. Results are presented as the average of 4–5 mice per group pooled from 2 independent infections. **B.** PAS staining of Carnoy's-fixed colorectal tissues of WT and *Muc2^−/−^* under uninfected or *C. rodentium*-infected (6 DPI) conditions. Very little mucin staining (magenta, arrows) can be seen in the epithelium or lumens of uninfected or infected *Muc2^−/−^* prior to or during infection. Results are representative of at least 3 independent infections with 2–3 mice per group. Original magnification  = 100×. Scale bar  = 100 µm.
(4.68 MB TIF)
