# Supplementary figures and images for: Muc2 Protects against Lethal Infectious Colitis by Disassociating Pathogenic and Commensal Bacteria from the Colonic Mucosa
Source: PLoS Pathog. 2010 May 13;6(5):e1000902. doi: 10.1371/journal.ppat.1000902 (PMC2869315; doi:10.1371/journal.ppat.1000902)

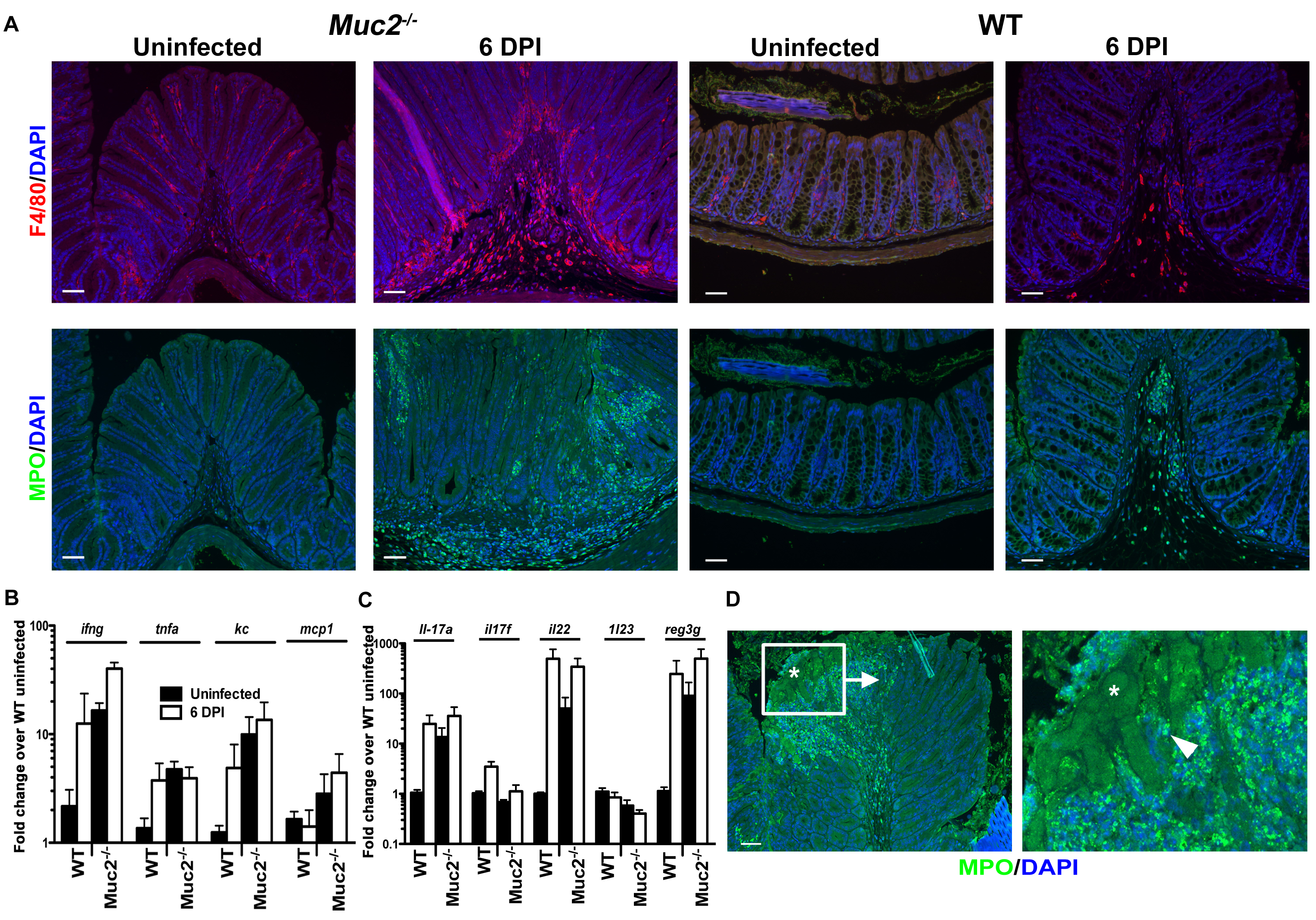

Supplement: Figure S1 — Characterization of the inflammatory cell infiltrate within the colons of C. rodentium-infected WT and Muc2−/− mice. A. Immunostaining for infiltrating macrophages via F4/80 staining (top panels) and neutrophils via MPO staining (bottom panels) in descending colons of WT and Muc2−/− and mice. Original magnification = 200×. Scale Bar = 50 µm. B. Quantitative PCR analysis of pro-inflammatory chemokines and cytokines in the descending colons of WT and Muc2−/− mice at 6 DPI compared to their respective uninfected controls. Results averaged from 3 independent infections, with n = 2–4 mice per group. Error bars = SEM. C. Quantitative PCR analysis of genes that are associated with host-susceptibility to C. rodentium in the colons of WT and Muc2−/− mice at 6 DPI. Results are averaged from 4–5 mice per group, pooled from 2 independent infections. Error Bars = SEM. D. MPO staining as above in an ulcerated region of an infected Muc2−/− mouse, showing a dense population of neutrophils in direct contact with a large microcolony of C. rodentium (white asterisk, C. rodentium aggregate; arrowhead, MPO positive cell in indirect contact with the microcolony). Original magnification = 200×. Scale Bar = 50 µm. (8.58 MB TIF) [file ppat.1000902.s001.tif]

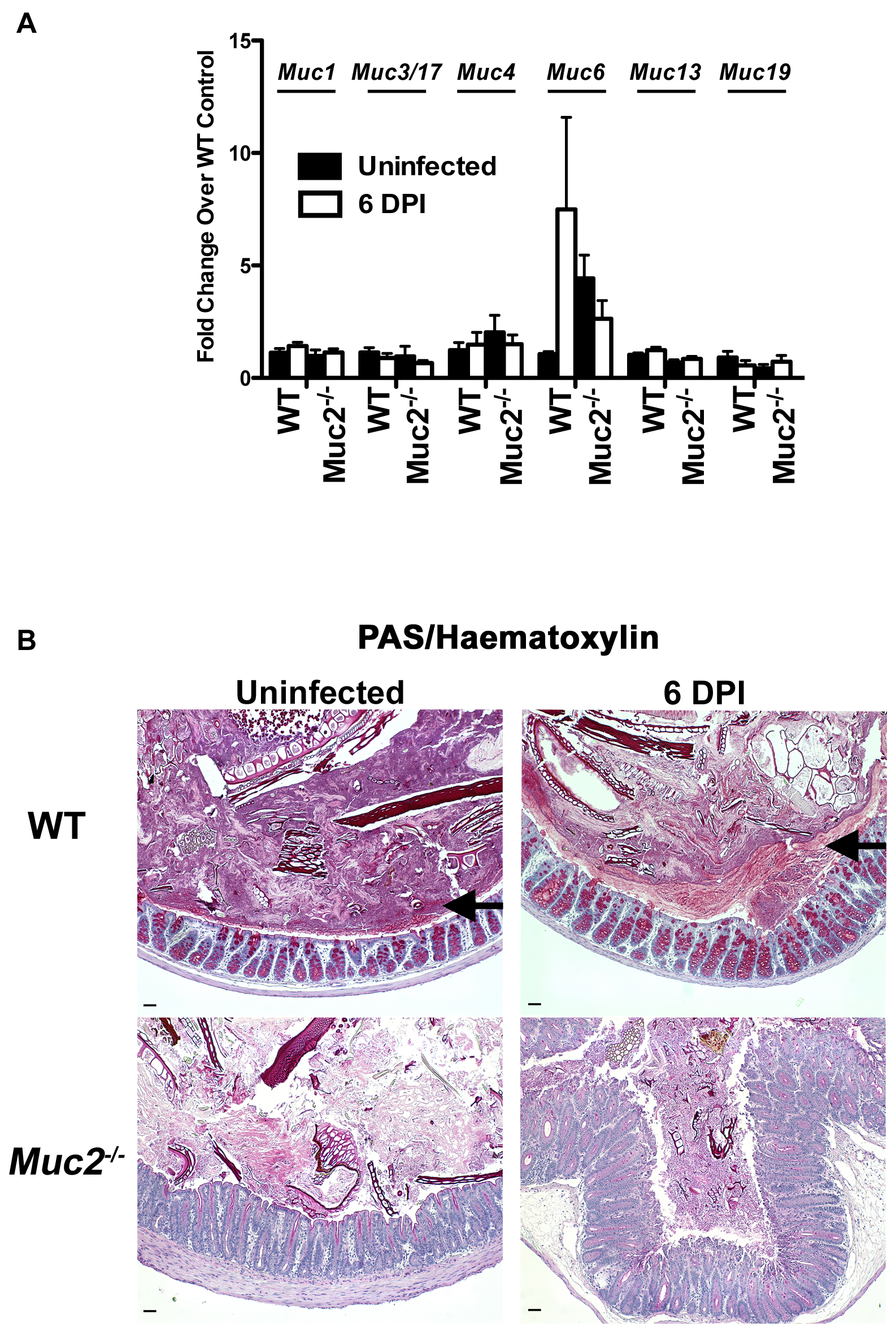

Supplement: Figure S2 — Analysis of Muc family gene expression and overall mucin content in colorectal tissues of uninfected or C. rodentium-infected WT and Muc2−/− mice. A. Quantitative PCR analysis of expression of genes encoding various Muc family members in the rectal tissues of WT and Muc2−/− mice under uninfected or infected (6 DPI) conditions. Results are presented as the average of 4–5 mice per group pooled from 2 independent infections. B. PAS staining of Carnoy's-fixed colorectal tissues of WT and Muc2−/− under uninfected or C. rodentium-infected (6 DPI) conditions. Very little mucin staining (magenta, arrows) can be seen in the epithelium or lumens of uninfected or infected Muc2−/− prior to or during infection. Results are representative of at least 3 independent infections with 2–3 mice per group. Original magnification = 100×. Scale bar = 100 µm. (4.68 MB TIF) [file ppat.1000902.s002.tif]
